# Enhanced Photocatalytic Activity and Stability in Hydrogen Evolution of Mo_6_ Iodide Clusters Supported on Graphene Oxide

**DOI:** 10.3390/nano10071259

**Published:** 2020-06-28

**Authors:** Marta Puche, Rocío García-Aboal, Maxim A. Mikhaylov, Maxim N. Sokolov, Pedro Atienzar, Marta Feliz

**Affiliations:** 1 Instituto de Tecnología Química, Universitat Politècnica de València-Consejo Superior de Investigaciones Científicas, Avenida de los Naranjos s/n, 46022 Valencia, Spain; mpuche@itq.upv.es (M.P.); rogarab@itq.upv.es (R.G.-A.); pedatcor@itq.upv.es (P.A.); 2 Nikolaev Institute of Inorganic Chemistry, Siberian Branch of the Russian Academy of Sciences, 3 Acad. Lavrentiev Ave., Novosibirsk 630090, Russia; mikhajlovmaks@yandex.ru (M.A.M.); caesar@niic.nsc.ru (M.N.S.)

**Keywords:** metal cluster, molybdenum, graphene oxide, nanocomposite, photocatalysis, hydrogen generation

## Abstract

Catalytic properties of the cluster compound (TBA)_2_[Mo_6_I^i^_8_(O_2_CCH_3_)^a^_6_] (TBA = tetrabutylammonium) and a new hybrid material (TBA)_2_Mo_6_I^i^_8_@GO (GO = graphene oxide) in water photoreduction into molecular hydrogen were investigated. New hybrid material (TBA)_2_Mo_6_I^i^_8_@GO was prepared by coordinative immobilization of the (TBA)_2_[Mo_6_I^i^_8_(O_2_CCH_3_)^a^_6_] onto GO sheets and characterized by spectroscopic, analytical, and morphological techniques. Liquid and, for the first time, gas phase conditions were chosen for catalytic experiments under UV–Vis irradiation. In liquid water, optimal H_2_ production yields were obtained after using (TBA)_2_[Mo_6_I^i^_8_(O_2_CCH_3_)^a^_6_] and (TBA)_2_Mo_6_I^i^_8_@GO) catalysts after 5 h of irradiation of liquid water. Despite these remarkable catalytic performances, “liquid-phase” catalytic systems have serious drawbacks: the cluster anion evolves to less active cluster species with partial hydrolytic decomposition, and the nanocomposite completely decays in the process. Vapor water photoreduction showed lower catalytic performance but offers more advantages in terms of cluster stability, even after longer radiation exposure times and recyclability of both catalysts. The turnover frequency (TOF) of (TBA)_2_Mo_6_I^i^_8_@GO is three times higher than that of the microcrystalline (TBA)_2_[Mo_6_I^i^_8_(O_2_CCH_3_)^a^_6_], in agreement with the better accessibility of catalytic cluster sites for water molecules in the gas phase. This bodes well for the possibility of creating {Mo_6_I_8_}^4+^-based materials as catalysts in hydrogen production technology from water vapor.

## 1. Introduction

**Figure 1:**
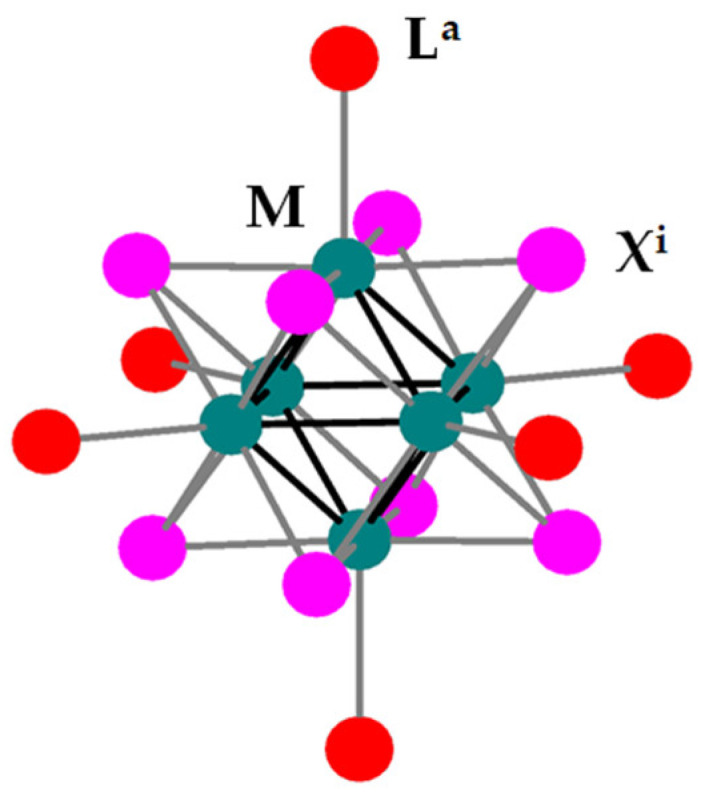
General representation of a [M_6_X^i^_8_L^a^_6_] (M = Mo, W; X^i^ = Cl, Br, I; L^a^ = apical ligand) cluster unit.

Dihydrogen production from water by sunlight (hydrogen evolution reaction, HER) is one of the most satisfactory ways of sustaining worldwide energy production and solving the looming environmental crisis [1,2,3]. The key challenge in pursuing this goal is to develop low-cost, stable, and efficient photocatalysts [4,5]. As molybdenum (and tungsten) is sufficiently cheap and abundant, their compounds constitute viable alternatives to costly noble metal-based luminophores, not to mention the environmentally hazardous lead-based hybrid perovskites and cadmium containing quantum dots [6,7,8]. Octahedral halide-bridged cluster compounds of Mo(II) and W(II), of the general type [M_6_X^i^_8_L^a^_6_] (M = Mo, W; X^i^ = Cl, Br, I (bridging or “inner”); L^a^ = organic/inorganic ligand (terminal or “apical”, see Figure 1) show remarkable photoluminescence properties and emit red light in high quantum yields, which makes them particularly attractive in the design of functional hybrid nanomaterials [9] with potential applications in optoelectronic [10,11,12,13,14,15,16,17,18], lighting [19], hydrogen storage [20], biomedicine [21,22,23,24,25,26,27,28,29], catalysis [30,31], and photocatalysis [32,33,34,35,36,37,38,39].

Among the [M_6_X^i^_8_L^a^_6_] clusters, the photoluminescence properties of the M_6_ iodide (X^i^ = I) clusters are superior to their bromide and especially chloride analogues in terms of quantum yield and phosphorescence time [40,41,42,43,44,45,46,47,48]. The combination of the {M_6_I^i^_8_}^4+^ cluster core with strongly electronegative O-donor ligands such as carboxylates, sulfonates, nitrates, or phosphonates in apical positions seems crucial to obtain from good to excellent emitters. One paradigmatic example is the (TBA)_2_[Mo_6_I^i^_8_(O_2_CC_3_F_7_)^a^_6_] cluster, which emits red phosphorescence with record quantum yield (X = Cl, *Φ*_em_ < 0.01; X = Br, *Φ*_em_ = 0.36; X = I, *Φ*_em_ = 0.59), unrivalled by almost any other known {Mo_6_I^i^_8_}^4+^ cluster [41]. These compounds bearing O-donor ligands have been developed during the last decade [49], and despite their outstanding photophysical properties, their applications in photocatalytic transformations remains scarce [35,37]. In a recent paper, the highly emissive [Mo_6_I^i^_8_(O_2_CC_2_F_5_)^a^_6_]^2−^ anion, combined with a bifunctional pyrene-imidazolium counterion, is supported via supramolecular anchoring onto graphene surfaces, and the resulting hybrid material (Py_2_Mo@Gene) combines the emission abilities of pyrene and cluster moieties to the electronic conduction efficiency of graphene [37]. HER studies show that this association induces a synergetic effect between graphene and the hybrid cluster complex, enhancing the photocatalytic conversion compared to that produced separately by clusters or graphene.

Other kinds of graphenic supports such as GO have been used to anchor octahedral molybdenum clusters on their surfaces. GO is a promising support material for metal catalysis due to its high surface area, good dispersion and distribution of metal active centers, excellent stability, high mechanical strength, electrical conductivity, and photocatalytic properties [50,51,52,53,54,55]. GO is formed by decorated graphene sheets with oxygen functional groups, and is an ideal processable form of graphene that can yield stable dispersions in various solvents [56,57]. The high number of oxygen-donor anchoring groups facilitates the immobilization and enhances the adsorption performance of metal catalysts [58,59,60]. Use of graphene oxide as a support has been reported in catalytic processes such as water or oxygen reduction reactions, coupling reactions, hydrodeoxygenation reactions, and, among other biomass conversion reactions [61,62,63]. Previous works have shown that the covalent grafting of {Mo_6_Br^i^_8_}^4+^ cluster core complexes on the surface of GO sheets enhances the stability of the cluster active sites for catalytic reactions to afford good catalysts for hydrogen generation from water [36], carbon dioxide transformations into valuable chemicals [34,64], and degradation of organic pollutants [33]. Our interest in the stabilization of the cluster active species in the photocatalytic hydrogen generation from water came from the formation of cluster decomposition species in solution, which was attributed to the effect of light and to the basicity of the media [36]. Actually, hexanuclear molybdenum clusters coordinated to halogens have been reported to decompose into Mo^III^ hydroxide in hot alkaline solutions [65]. The non-innocent behavior of the solvent in the preservation of the integrity of the {Mo_6_X^i^_8_}^4+^ cluster core compounds has encouraged us to explore their catalytic reactivity with gas phase reactants.

Gas phase photocatalytic reactions have been poorly explored in comparison to those performed in aqueous phase, and they constitute a promising alternative to the latter [66]. In some cases, the major economical drawback is the high temperature required for hydrogen production [67]. In the last decade, some authors have proven the photochemical water splitting in soft conditions (below 55 °C) [68]. The main advantage of this vapor phase photochemical water activation is that the stability of the catalyst is generally secured, and the recovery of the catalyst is assured. In addition, most of the liquid sacrificial compounds vaporize, and they can be made easily recoverable by condensation.

In this work, we studied the photocatalytic activity and stability of the octahedral molybdenum (II) iodide cluster compound (TBA)_2_[Mo_6_I^i^_8_(O_2_CCH_3_)^a^_6_] and of the hybrid (TBA)_2_Mo_6_I^i^_8_@GO nanocomposite in the production of hydrogen as one of the most representative reactions in the field of renewable energy. The cluster compound (TBA)_2_[Mo_6_I^i^_8_(O_2_CCH_3_)^a^_6_] was chosen for two main reasons: (i) its good photophysical properties (*Φ*_em_ = 0.48), as reported by Sokolov et al. [46], and (ii) its composition makes it a good candidate for immobilization onto GO sheets by simple substitution of the carboxylate ligands by the oxygen functionalities of the graphene surface. In fact, it was recently reported that the apical acetate ligands can be replaced by other carboxylates such as isonicotinate ligands [69] where the coordinative anchoring of the highly luminescent {Mo_6_I^i^_8_}^4+^ cluster cores through the GO functionalities would closely imitate the molybdenum–acetate bonds in [Mo_6_I^i^_8_(O_2_CCH_3_)^a^_6_]^2−^. Hence, we prepared (TBA)_2_Mo_6_I^i^_8_@GO by anchoring the {Mo_6_I^i^_8_}^4+^ cluster cores onto the surface of GO. Ligand exchange between the carboxylate apical groups and the oxygen functionalities of the GO nanosheets takes place in the immobilization process. This hybrid nanomaterial was characterized by means of Fourier transform infrared spectroscopy (FTIR), Raman, UV–Vis, photoluminescence, x-ray diffraction (XRD), high-resolution transmission electron microscopy (HR-TEM), inductively coupled plasma atomic emission spectrometry (ICP-AES), and combustion analysis. The resulting molecular and hybrid materials were studied in liquid phase photocatalytic water reduction into H_2_ under UV–Vis irradiation and, for the first time, they were proven to work for hydrogen generation employing only gas phase water. Catalytic vapor water photoreduction is presented as the optimal methodology to enhance the activity and stability of the {Mo_6_I^i^_8_}^4+^ cluster active sites.

## 2. Materials and Methods

### 2.1. Chemicals

Triethylamine (TEA), acetone, dimethylformamide (DMF), methanol, and anhydrous ethanol were obtained from commercial resources (Sigma-Aldrich, Darmstadt, Germany). Tetrahydrofuran (THF) and acetonitrile was dried and deoxygenated by passing these solvents through commercial columns of CuO, followed by alumina under a nitrogen atmosphere. Acetonitrile (HPLC grade) was used as the solvent for mass spectrometry analyses. For the photocatalytic reactions in solution, Milli-Q water, TEA, DMF, acetone, and methanol were deoxygenated by bubbling dry nitrogen for at least half an hour. GO was prepared from natural graphite by following an optimized procedure of the improved Hummer’s synthetic method [70,71]. The (TBA)_2_[Mo_6_I^i^_8_(O_2_CCH_3_)^a^_6_] compound was synthesized by the reported procedure [46].

### 2.2. Instrumentation

Combustion chemical analysis of the samples were carried out using a Fisons EA 1108-CHNS-O analyzer (ThermoFisher Scientific, Waltham, MA, USA). ICP-AES analyses for the determination of atomic molybdenum of the solid materials were performed after aqua regia digestion and the resulting solutions were measured in a Varian 715 spectrometer (Palo Alto, CA, USA). FTIR spectra were measured on KBr pellets with a Nicolet 8700 Thermo spectrometer (ThermoFisher Scientific, Waltham, MA, USA). The Raman spectra were acquired from solid samples previously deposited onto aluminum or quartz wafers, indistinctively, using a “Reflex” Renishaw spectrometer (Wotton-under-Edge, UK) equipped with an Olympus microscope. The exciting wavelength was 514 nm of an Ar^+^ ion laser. The laser power on the sample was ~10–25 mW and a total of 20 acquisitions were taken for each spectra. UV-vis spectra were recorded at 20 °C with a Varian Cary 50 Conc spectrophotometer (Palo Alto, CA, USA) equipped with 10 × 10 mm quartz cuvettes. The UV–Vis spectra of (TBA)_2_[Mo_6_I^i^_8_(O_2_CCH_3_)^a^_6_] were recorded in CH_3_CN and the samples taken before and after heterogeneous catalytic reaction were registered in CH_2_Cl_2_; the spectra of (TBA)_2_Mo_6_I^i^_8_@GO and GO were recorded from a stable suspension of the material dispersed in Milli-Q water. Steady-state photoluminescence measurements of (TBA)_2_[Mo_6_I^i^_8_(O_2_CCH_3_)^a^_6_] were conducted at 20 °C throughout the study. The powdered samples of the molecular compound were placed between two nonfluorescent glass plates. A solution of (TBA)_2_[Mo_6_I^i^_8_(O_2_CCH_3_)^a^_6_] in CH_3_CN in 10 × 10 mm quartz cuvettes was deaerated by purging with an Ar gas stream for 30 min, and then the cuvettes were sealed. The sample was excited with 355 nm laser pulses (6 ns duration, LOTIS TII, LS-2137/3). Corrected emission spectra were recorded on a red-sensitive multichannel photodetector (Hamamatsu Photonics, PMA-11, Naka-ku, Hamamatsu City, Japan). Photoluminescence measurements of (TBA)_2_Mo_6_I^i^_8_@GO and of the experiment based on the addition of GO suspensions to a (TBA)_2_[Mo_6_I^i^_8_(O_2_CCH_3_)^a^_6_] solution in DMF were recorded in 10 × 10 mm quartz cuvettes at 20 °C and by continuous purging with an N_2_ gas stream. The samples were excited with 345 nm in a Photon Technology International (PTI) 220B spectrofluorimeter (Horiba, Minami-ku, Kyoto, Japan) with Xe arc lamp light excitation and a Czerny–Turner monochromator, coupled to a photomultiplier. Electrospray ionization mass spectrometry (ESI-MS) was recorded with a Xevo Q ToF (quadrupole Time-of-Flight) spectrometer and with a QTof Premier (quadrupole-hexapole-ToF) equipped with an orthogonal Z-spray electrospray interface (Waters, Manchester, UK), and liquid samples were solved in acetonitrile before direct infusion. A capillary voltage of 2.4 kV was used in the positive and negative scan modes, respectively, and the cone voltage was set to 20 V. Nitrogen was employed as drying and nebulizing gas. The powder XRD data of (TBA)_2_[Mo_6_I^i^_8_(O_2_CCH_3_)^a^_6_] (thickness of a thin even layer of the sample of 100 μm) were collected on a Shimadzu XRD-7000 diffractometer (Nakagyo-ku, Kyoto, Japan) with CuKα radiation, OneSight linear detector, 2*θ* range 5–70° with steps 0.0143° 2*θ* and 2 s accumulation at room temperature. Powder XRD patterns of (TBA)_2_Mo_6_I^i^_8_@GO and GO were obtained by using a Philips X′Pert diffractometer (Philips, Amsterdam, The Netherlands) and copper radiation (CuKα = 1.541178 Å). Samples for HR-TEM were ultrasonically dispersed in Milli-Q water and transferred into carbon coated copper grids. HR-TEM images were recorded by using a JEOL JEM2100F microscope (Akishima, Tokyo, Japan) operating at 200 kV. The molybdenum and iodine contents of the (TBA)_2_Mo_6_I^i^_8_@GO sample were determined by using an energy-dispersive x-ray analysis (EDXA) system (Oxford Instruments) attached to a JEOL JEM2100F electronic microscope. An analysis of the porous structure of (TBA)_2_[Mo_6_I^i^_8_(O_2_CCH_3_)^a^_6_] was performed by a nitrogen adsorption technique using Quantochrome’s Autosorb iQ (Boynton Beach, FL, USA) at 77 K. Initially, the compound was first activated in dynamic vacuum using the standard “outgas” option of the equipment at 40 °C during 6 h. N_2_ adsorption−desorption isotherms were measured within the range of relative pressures of 10^−3^ to 0.997. The specific surface area was calculated from the data obtained on the basis of the conventional Brunauer–Emmett–Teller (BET) model.

### 2.3. Single-Crystal Isolation and X-ray Data Collection

Suitable red polyhedral (truncated tetrahedron) crystals for x-ray studies of [Mo_6_I^i^_8_(OH)^a^_4_(H_2_O)^a^_2_]·2H_2_O were grown by slow crystallization in a water/acetone/TEA mixture. Namely, once the (TBA)_2_[Mo_6_I*^i^*_8_(O_2_CCH_3_)*^a^*_6_] precursor was solubilized in water/acetone/TEA (50/45/5% v/v), it was subjected to vacuum during 2 h at room temperature and a mixture of red polyhedral and laminar crystals were formed in a transparent solution after overnight. The crystals obtained were washed twice with acetone and once with Milli-Q water, and the laminar crystals disappeared. The colored liquid phases were identified by UV–Vis, indicating the presence of the unreacted precursor complex. The isolated polyhedral crystals remained stable under air for at least four months and further assays confirmed that they were not soluble in water, acetone, methanol, DMSO, or DMF.

Diffraction data for [Mo_6_I^i^_8_(OH)^a^_4_(H_2_O)^a^_2_]·2H_2_O were collected on an Agilent Supernova diffractometer (Yarnton, UK) equipped with an Atlas CCD detector using CuKα radiation (*λ* = 1.54184 Å). Numerical absorption correction was based on Gaussian integration over a multifaceted crystal model [72]. Empirical absorption correction was conducted using spherical harmonics, implemented in the SCALE3 ABSPACK scaling algorithm.

### 2.4. Synthesis and Characterization of (TBA)_2_Mo_6_I^i^_8_@GO

A GO suspension was prepared by exfoliation of the GO (100 mg) by sonication with an ultrasound source (400 W, Branson ultrasonic bath, Brookfield, CT, USA) in THF (150 mL) for 1 h under N_2_. The GO suspension was added to a solution of (TBA)_2_[Mo_6_I^i^_8_(O_2_CCH_3_)^a^_6_] (74 mg, 0.031 mmol) in ethanol (50 mL) and the resulting mixture was heated to 60 °C during 16 h and magnetically stirred under N_2_ using standard Schlenk techniques. The solid product was separated from the solution by filtration, washed several times with ethanol, and dried under vacuum to provide 127 mg of dark brown-colored product identified as (TBA)_2_Mo_6_I^i^_8_@GO. The amount of molybdenum present in the sample (1.55 wt%) was determined by ICP-AES analysis. This material was characterized by FTIR, Raman, UV–Vis, XRD, HR-TEM, and combustion analysis (C 47.06%, H 1.77%, N 0.11%) techniques. This material was stored in a desiccator [73]. Our attempts to determine the surface area of the (TBA)_2_Mo_6_I^i^_8_@GO and GO materials by using the BET isotherm by N_2_ adsorption were unsuccessful because of the high dispersibility and low values obtained. The surface area of GO was previously determined (736.6 m^2^/g) by the adsorption of methylene blue (MB) [74]; nevertheless, this methodology was not considered suitable for the measurement of the surface area of (TBA)_2_Mo_6_I^i^_8_@GO, since the MB cation could be involved in the counterion exchange of the cluster-supported species and no optimal adsorption to the graphenic surface would be achieved.

### 2.5. Photocatalytic H_2_ Evolution Procedure

**Figure 2:**
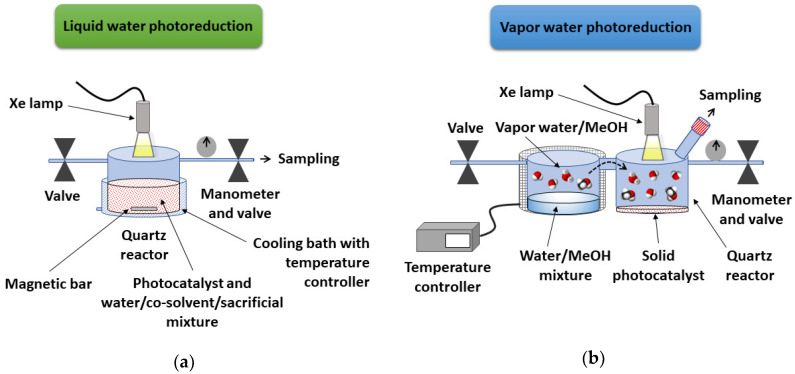
Experimental setups for liquid water (**a**) and vapor water (**b**) catalytic photoreductions.

UV–Vis irradiations were carried out with a spot light Hamamatzu Xe lamp (Lightnincure LC8 model, 800–200 nm, 1000 W/m^2^, fiber optic light guide with a spot size of 0.5 cm). For the photocatalytic reactions performed in the presence of an aqueous solution, the chosen photoreactor was a cylindrical Pyrex vessel with a total volume of 55 mL and was 140 mm in diameter, with an inlet and outlet with independent valves equipped with a manometer to determine the pressure. Figure 2a illustrates the experimental setup for liquid water photoreduction. In a typical reaction, the photoreactor was initially charged with 15 mL solvent mixture of Milli-Q water (with or without an organic co-solvent) and TEA (5% v/v) or methanol (30% v/v) as electron donors. The whole system was purged with a nitrogen flow for 30 min. The amount of photocatalyst (30 and 0.54 mg of (TBA)_2_[Mo_6_I^i^_8_(O_2_CCH_3_)^a^_6_] for standard and comparative studies, respectively; 30 and 11 mg of (TBA)_2_Mo_6_I^i^_8_@GO for standard and comparative studies, respectively) was added to the solution under a N_2_ atmosphere, and the photoreactor was re-purged (10 min) under vigorous magnetic stirring to ensure the absence of oxygen in the system. The reaction vessel was sealed and pressurized with N_2_ up to 0.5 bar. This was kept 1.5 cm away from the light source and was immersed into a thermostatic bath at 25 °C during irradiation (5 h of standard time). The gas phase samples (100 μL) were collected with a Samplelock Hamilton syringe. The amount of hydrogen evolved in the photoreactor during irradiation was determined by gas chromatography (GC) with an Agilent 6850 GC system (Santa Clara, CA, USA) equipped with a bonded polystyrene–divinylbenzene HP-PLOT Q column (30 m length, 0.53 mm inner diameter, 40 μm film thickness; Agilent J&W) and a thermal conductivity detector (TCD). Helium was the carrier gas, and the flow rate was set to 5 mL/min. The temperatures of the injector and detector in GC analysis were 53 and 220 °C, respectively, and the isothermal oven temperature profile was set to 50 °C.

For the photocatalytic reactions carried out in the presence of aqueous mixtures in vapor phase, the photoreactor was a double cylindrical quartz reactor (110 mL of total volume) in which the two vessels were connected with a quartz bridge (2 cm length, see Figure S1). Figure 2b illustrates the experimental layout for vapor water photoreduction. Water (30 mL Milli-Q water) and the sacrificial electron donor (methanol, 30% v/v) were loaded into the reactor vessel (a) and purged with Ar (30 min). The photocatalyst (10 and 5 mg of (TBA)_2_[Mo_6_I^i^_8_(O_2_CCH_3_)^a^_6_]; 10 and 30 mg of (TBA)_2_Mo_6_I^i^_8_@GO) was loaded into the reactor vessel (b). The reactor was sealed and pressurized with argon up to 0.5 bar and connected to an electrical heating ribbon that allowed the reactor vessel (a) to be heated to 70 °C in order to achieve the evaporation of the water/sacrificial mixture. The vessel (a) was irradiated for 24 h (standard irradiation time). The H_2_ generation was determined by an Agilent 490 Micro GC equipped with a molecular-sieve-coated CP-Molsieve 5Å column, Agilent J&W) and a thermal conductivity detector (TCD). Ar was taken as the carrier gas, and the flow rate was set to 5 mL·min^−1^. The temperatures of the injector and detector in GC analysis were 110 and 220 °C, respectively, and the isothermal oven temperature profile was set to 62 °C with an initial column pressure of 15 psi.

The hydrogen peak area was converted to the corresponding concentrations, based on the standard calibration curve. The moles of hydrogen generated were calculated by using the ideal gas law (*n* = *PV*/*RT*). Control experiments were performed, one under UV–Vis irradiation without a photocatalyst, and another under dark conditions using the cluster materials at the standard experimental conditions. In the reactions performed with water in the vapor phase, the use of TEA as a sacrificial electron donor was discarded because the control reactions, in the absence of the molybdenum photocatalyst, afforded methane as a subproduct due to the decomposition of TEA in the reaction conditions. In contrast, during the photocatalytic experiments executed with the halogenated hexanuclear molybdenum complexes described in this work, only hydrogen, atmospheric nitrogen, and oxygen were detected by GC-TCD analyses, independently of the sacrificial donor used. Measurements from the control experiments showed the detection of the atmospheric gases exclusively, which confirmed the selective water to hydrogen photoreduction.

Reuse experiments of the (TBA)_2_[Mo_6_I^i^_8_(O_2_CCH_3_)^a^_6_] complex and (TBA)_2_Mo_6_I^i^_8_@GO nanocomposite were done under standard catalytic conditions after recovering the solid material by evaporation (cluster complex) or filtration (hybrid material) under vacuum.

## 3. Results and Discussion

### 3.1. Photocatalytic Activity of the (TBA)_2_[Mo_6_I^i^_8_(O_2_CCH_3_)^a^_6_] Compound in the Photoreduction of Water in Liquid Phase

A comparative study of the photocatalytic activity of the (TBA)_2_[Mo_6_I^i^_8_(O_2_CCH_3_)^a^_6_] cluster compound in molecular hydrogen generation from liquid water was accomplished in the presence of an electron donor under UV–Vis irradiation and different reaction conditions. The performance of the catalyst was evaluated in terms of H_2_ yield after irradiation time. Initially, the (TBA)_2_[Mo_6_I^i^_8_(O_2_CCH_3_)^a^_6_] cluster compound was tested in the HER process using a sacrificial agent (methanol or TEA) under UV–Vis irradiation. We obtained 217 μmol/g_cat_ of H_2_ in the presence of TEA, but none with methanol. These results were unexpected, since better results (3205 μmol/g_cat_) were reported for the (TBA)_2_[Mo_6_Br^i^_8_F^a^_6_] cluster analogue under identical catalytic conditions [36]. With the purpose of optimizing and enhancing the catalytic process, we planned to use suitable water/organic solvent mixtures to ensure the rising of the concentration of the cluster catalyst in solution to achieve homogeneous conditions, and to stabilize the anionic [Mo_6_I^i^_8_(O_2_CCH_3_)^a^_6_]^2-^ cluster from the (TBA)_2_[Mo_6_I^i^_8_(O_2_CCH_3_)^a^_6_] ionic pair. The effects on the hydrogen evolution were studied following systematic changes in: (i) the nature of the organic solvent, and (ii) the fractional amount of water, always in the presence of TEA.

Acetonitrile, DMF, and acetone were chosen as suitable polar co-solvents due to their different dielectric constants, diffusion coefficients, and for the optimal solubility performance for the cluster catalyst, and were used in 65% v/v to ensure the homogeneous conditions. The hydrogen production yields obtained in the presence of acetonitrile or DMF were quite low (63 and 95 μmol/g_cat_, respectively), however, the catalytic system containing acetone was found to give the best results in terms of both slope (rate of hydrogen production) and maximum chemical yield (172 μmol/g_cat_ of H_2_, see Figure S2).

**Figure 3:**
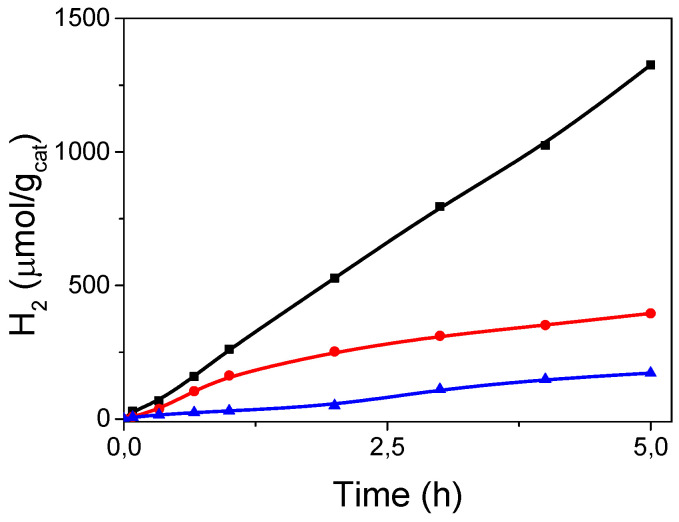
UV–Vis light driven hydrogen generation of (TBA)_2_[Mo_6_I^i^_8_(O_2_CCH_3_)^a^_6_] in aqueous solution containing TEA, water in 50% (black line), 40% (red line), and 30% (blue line) v/v and acetone.

We found that the addition of a 45% v/v of acetone was enough to ensure strict homogeneous catalytic conditions. Therefore, we increased the content of water to 40% and 50% v/v and the hydrogen-evolving performance improved to 396 and 1326 μmol/g_cat_, respectively (Figure 3). Under these optimized conditions, the photocatalytic H_2_ evolution rate increased up to 265 μmol/h·g_cat_ in the first 5 h of irradiation. This evolution rate is higher than the value reported for the analogous Py_2_[Mo_6_I^i^_8_(O_2_CC_2_F_5_)^a^_6_] (Py_2_Mo, Py = (1-methyl-3-(4-(pyren-1-yl)butyl)-1H-imidazol-3-ium)) catalyst (17 μmol/h·g_cat_), for the same reaction time and under similar conditions [37], than the rates reported for other molybdenum cluster and nanoparticles (see Table S1).

Plotting the hydrogen generation against time shows the absence of an induction period and a production of 29 μmol/g_cat_ after 5 min reaction (Figure 3). The evolution rate at 5 min of irradiation (350 μmol/h·g_cat_), however, decreased linearly to 23% from 20 min to 5 h reaction time (Figure S3), revealing unfavorable evolution of the active starting [Mo_6_I^i^_8_(O_2_CCH_3_)^a^_6_]^2−^ complex in solution. We performed an ESI-MS analysis of three liquid samples taken at *t* = 0, 60, and 105 min of irradiation. The results indicate that the complex evolves to [Mo_6_I^i^_8_(O_2_CH_3_)^a^_5_(OH)]^2−^ and [Mo_6_I^i^_8_(O_2_CH_3_)^a^_4_(OH)_2_]^2−^ species (Figure S4) after 105 min reaction time, and these species are generated in situ under photocatalytic conditions. A red microcrystalline precipitate was identified as [Mo_6_I^i^_8_(OH)^a^_4_(H_2_O)^a^_2_]·2H_2_O, whose structure was also determined by single-crystal x-ray characterization (see Supplementary Materials and Figures S5–S7). Likewise behavior was reported by Feliz, Cordier et al. for the evolution of [Mo_6_Br^i^_8_F^a^_6_]^2−^ to [Mo_6_Br^i^_8_F^a^*_x_*(OH)^a^_6−*x*_]^2−^ (*x* = 6–0) species in water/TEA mixtures and under identical photochemical conditions [36]. In this work, the species generated in solution contributed to an enormous increase of the evolution rate. In contrast, the above-mentioned hydroxo {Mo_6_I_8_}^4+^ cluster core species produced under reaction conditions did not seem to participate in raising the efficiency of the starting complex at the same level. In addition, we observed a progressive solution color change from orange to brownish along with irradiation time, which suggests photodecomposition of the cluster catalyst. At the end of the reaction, the reaction mixture was evaporated, and the resulting solid was tested in a reuse experiment. The decrease of the catalytic activity confirmed the partial degradation of the cluster catalyst. The complete decomposition of the cluster species in solution was set by ESI-MS characterization (Figure S8) of a reaction sample after long radiation exposure (24 h).

All the above indicates that these catalytic reaction conditions promote the generation of the other active cluster species and accelerate the hydrolytic transformation of the cluster complex. Previous works have demonstrated that the covalent grafting of active octahedral molybdenum clusters onto the surface of GO sheets enhances the stability of the cluster core under catalytic conditions and converts the hybrid composite into a reusable catalyst [34,36,64]. Therefore, with the purpose to ensure the integrity of the octahedral cluster cores, we decided to develop a new hybrid material based on the {Mo_6_I^i^_8_}^4+^ cluster cores anchored onto GO surfaces and to apply it in the HER process.

### 3.2. Synthesis and Characterization of the (TBA)_2_Mo_6_I^i^_8_@GO Nanocomposite

The (TBA)_2_[Mo_6_I^i^_8_(O_2_CCH_3_)^a^_6_] compound contains exchangeable unidentate acetate ligands in apical positions, which can be selectively substituted by the carboxylate and/or alkoxy groups attached onto the graphene oxide surface. In previous works, the {Mo_6_Br^i^_8_}^4+^ cluster cores were successfully immobilized onto GO nanosheets via coordinative anchoring [34,36,64]. The A_2_[Mo_6_Br^i^_8_X^a^_6_] (A = Cs or TBA; X^a^ = F or Br) compounds were used as precursors for the preparation of hybrid GO-based materials. With the purpose of immobilizing the (TBA)_2_[Mo_6_I^i^_8_(O_2_CCH_3_)^a^_6_] cluster onto GO nanosheets in mind, a THF dispersion of GO and an ethanolic solution of (TBA)_2_[Mo_6_I^i^_8_(O_2_CCH_3_)^a^_6_] were mixed in a 3:1 volume ratio and kept for 16 h at 60 °C. The resulting composite material (TBA)_2_Mo_6_I^i^_8_@GO was isolated by filtration. The content of molybdenum (1.55 wt%) was confirmed by ICP-AES analysis. This material was thoroughly washed from the cluster precursor and characterized by Raman and FTIR spectroscopies, UV–Vis, x-ray diffraction, HR-TEM, EDX, and elemental analyses.

The carbon content lies in the 44–50% range characteristic of the GO-based materials, and the nitrogen percentage agrees with the presence of two TBA counterions per cluster unit in the (TBA)_2_Mo_6_I^i^_8_@GO hybrid material. The x-ray diffraction pattern shows a single diffraction peak at 2*θ* = 11.5°, associated with the characteristic (001) plane of the GO composites (Figure S9). No additional diffraction peaks associated to the molecular cluster compound were detected due to the low amount of supported cluster and the laminar structure of the composite. The UV–Vis spectrum of a (TBA)_2_Mo_6_I^i^_8_@GO suspension showed two characteristic absorption maxima at 300 (sh) and 236 (b) nm. These bands correspond to the n–π* and π−π* electronic transition modes in the GO, respectively, and the slight bathochromic shift of the second band with respect to the GO precursor (229 nm) is due to the contribution of the most intense absorption of the (TBA)_2_[Mo_6_I^i^_8_(O_2_CCH_3_)^a^_6_] compound at 258 nm. The photoluminescence study of the (TBA)_2_Mo_6_I^i^_8_@GO nanocomposite did not show the characteristic emission of the cluster compound at ca. 700 nm [46]. This silence can be due to a phosphorescence quenching of the cluster by means of the GO. In order to demonstrate this, we performed successive additions of a GO suspension (2.5 mg/L in DMF) to a solution of (TBA)_2_[Mo_6_I^i^_8_(O_2_CCH_3_)^a^_6_] (10^−5^ M in DMF). The cluster emission diminished after two successive additions of the GO suspension (Figure S10). This behavior is attributed to the quenching of the photoluminescence of the cluster compound by the GO material associated with the non-radioactive energy transfer from the cluster compound to the graphenic surface [75,76,77]. We have previously reported a similar quenching behavior by loading successive additions of a volume of a graphene suspension to a solution of [Mo_6_I^i^_8_(O_2_C_2_F_5_)^a^_6_]^2−^ complex [37].

**Figure 4:**
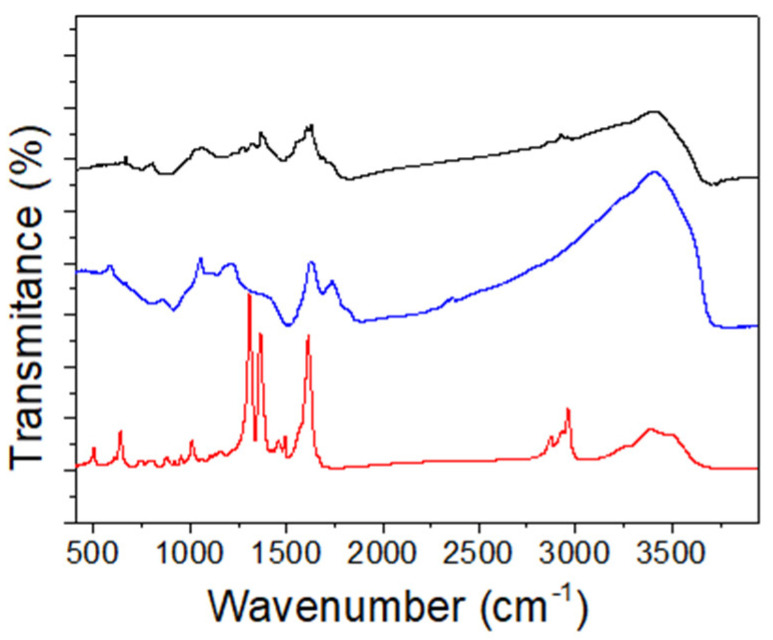
Fourier transform infrared (FTIR) spectra of (TBA)_2_Mo_6_I^i^_8_@GO (back line), GO (blue line) and (TBA)_2_[Mo_6_I^i^_8_(O_2_CCH_3_)^a^_6_] (red line).

The FTIR identification of (TBA)_2_Mo_6_I^i^_8_@GO (Figure 4) shows two strong characteristic C=O vibration bands at 1723 and 1627 cm^−1^, which correspond to the carbonyl and carboxylic/adsorbed water vibration bands, respectively, characteristic of the GO material. The representative C=O band of the molecular (TBA)_2_[Mo_6_I^i^_8_(O_2_CCH_3_)_6_] starting compound at 1616 cm^−1^ disappears after the cluster immobilization [46], and a new band appears instead at 1603 cm^−1^. This band is attributed to the interaction of the hexametallic clusters with the carboxylate functionalities of the graphene support. In addition, the epoxy/hydroxyl vibration region of the GO appears distorted in comparison to pure GO vibrations: the band characteristic of the C–OH graphene vibrations of GO at 1210 cm^−1^ is hypsochromic to a band at 1272 cm^−1^. The epoxy region is altered, because the intense band (1060 cm^−1^) assigned to the C–O–C vibration of GO disappears, and new bands at 1068 and 1028 cm^−1^ appear. These bands are to be assigned to new Mo(O–C) vibrations, which fit into the vibration window of the carboxylate and alkoxy apical groups attached to the {Mo_6_X^i^_8_}^4+^ (X^i^ = Cl, Br or I) cluster cores [46,78,79]. It is worth noting that our previously reported (TBA)_2_Mo_6_Br^i^_8_F^a^_6_@GO nanocomposite shows changes uniquely in the epoxy/hydroxyl IR window, thus confirming that the hydroxyl groups of the GO support are involved selectively in the cluster immobilization of (TBA)_2_[Mo_6_Br^i^_8_F^a^_6_] onto GO nanosheets [36]. Additional bands at 1369 and 1556 cm^−1^ were detected and assigned to the C–N and C–C vibrations, respectively, which confirms the presence of the TBA counterions. These results unequivocally indicate that the ligand exchange between the carboxylate groups of the molecular (TBA)_2_[Mo_6_I^i^_8_(O_2_CCH_3_)^a^_6_] cluster by the carboxylic and hydroxylic groups of the GO surface and, as a consequence, the coordinative immobilization of the {Mo_6_I^i^_8_}^4+^ cluster cores takes place in the reaction conditions.

**Figure 5:**
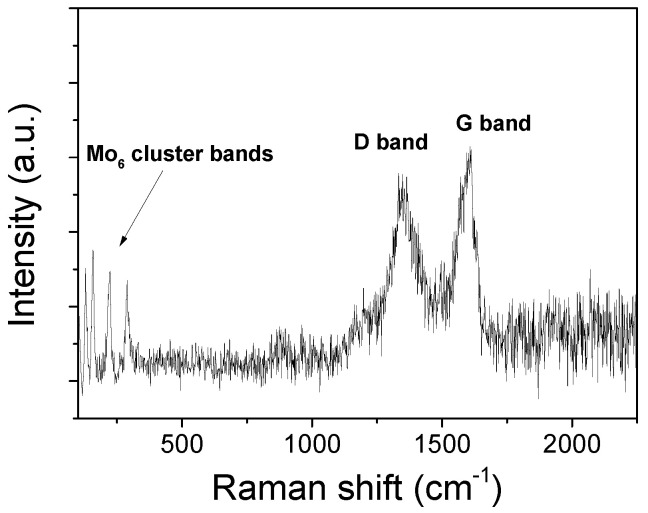
Raman spectrum of (TBA)_2_Mo_6_I^i^_8_@GO under 514 nm excitation.

The Raman spectroscopy confirms the retention of the {Mo_6_I^i^_8_}^4+^ cluster core in the (TBA)_2_Mo_6_I^i^_8_@GO nanohybrid. The Raman spectrum of (TBA)_2_Mo_6_I^i^_8_@GO (Figure 5) shows intense bands with Raman shifts at 130, 159, 224, and 284 cm^−1^, which are reasonably close to the majority of most of the molecular cluster Raman shifts (153 (m), 166 (m), 170 (m), 220 (int), and 285 (m) cm^−1^). These bands are assigned to the Mo–Mo, Mo–I, and Mo–O vibrations of the cluster [80]. At higher Raman frequencies, two bands appear at 1350 and 1600 cm^−1^, which correspond to the respective D and G bands characteristic of the GO.

**Figure 6:**
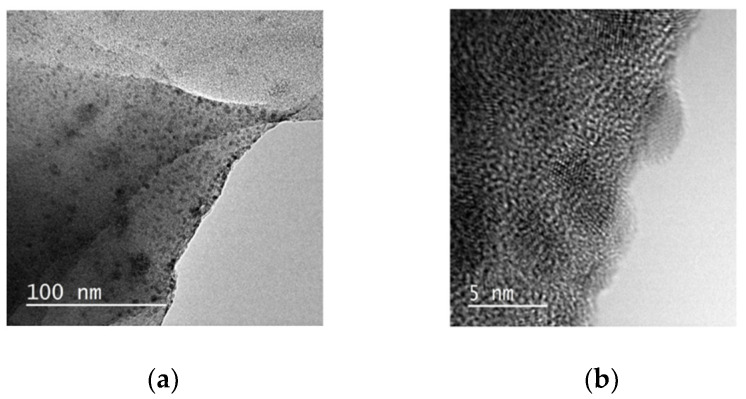
HR-TEM images of (TBA)_2_Mo_6_I^i^_8_@GO at 100 (**a**) and 5 nm (**b**) scale, registered at 200 kV.

The HR-TEM images of (TBA)_2_Mo_6_I^i^_8_@GO (Figure 6) show similar lamellar morphologies to those observed for the GO precursor [70], which provides an estimate of the high surface area of this nanocomposite, and an homogeneous dispersion of crystalline nanoparticles (3–5 nm), which are associated with aggregated {Mo_6_I^i^_8_}^4+^ cluster cores on the surfaces and mostly on the borders of GO. These results suggest that the anchoring of the cluster takes place through the hydroxylic and the carboxylic groups of GO located on the edges of the sheets, according to the Lerf–Klinowski and Ajayan models [56,81]. The supported nanoparticles show a linear distribution of molybdenum atoms with a distance of 1.3 Å between the planes crystallographically defined according to the single-crystal x-ray results. Dark field quantification of the sample by EDXA of (TBA)_2_Mo_6_I^i^_8_@GO (Figure S11) yields the atomic content of molybdenum and iodine of 1.12 and 1.59%, respectively. The experimental atomic ratio of I/Mo = 1.4 is in agreement with the calculated value of (1.3) for the {Mo_6_I^i^_8_}^4+^ cluster core. This confirms, again, that intact {Mo_6_I^i^_8_} clusters are supported onto the GO nanosheets and that the cluster core remains intact after the immobilization.

### 3.3. Catalytic Properties of (TBA)_2_Mo_6_I^i^_8_@GO in the Photoreduction of Liquid Phase Water

The hybrid (TBA)_2_Mo_6_I^i^_8_@GO material was studied in the photocatalytic water reduction in the presence of TEA in a water/acetone (50/45% v/v) mixture. In this case, the yield achieved was 291 μmol/g_cat_ of H_2_ and, actually, the hybrid material showed 78% lesser catalytic activity than the molecular compound under identical photochemical conditions. Additional catalytic tests were done using (TBA)_2_Mo_6_I^i^_8_@GO, GO and the equimolar amount of the molecular complex as catalysts (Figure S12). These showed an enhancement of the catalytic activity of (TBA)_2_Mo_6_I^i^_8_@GO compared to the GO. The catalytic activity of (TBA)_2_Mo_6_I^i^_8_@GO can be mainly attributed to the immobilized molybdenum cluster active sites, since no other synergetic effect between Mo centers and GO was observed.

The (TBA)_2_Mo_6_I^i^_8_@GO catalyst was recovered and reused, and the catalytic yield dropped by 93% (Figure S13). The activity loss is ascribed to the decomposition of the hybrid catalyst under reaction conditions. To ascertain the leaching of the metal, we analyzed the recovered catalyst by ICP-AES. The value of Mo in the recovered catalyst was found to be only one-third (0.50 wt%) of that in the fresh catalyst (1.55 wt%). These results indicate that leaching indeed occurred during the reaction and the catalyst was not actually heterogeneous under reaction conditions. We attributed the poor stability of the (TBA)_2_Mo_6_I^i^_8_@GO material, dispersed in aqueous solutions, to one or two of the following points: (i) the use of TEA as sacrificial, since TEA could affect the nature of the hybrid Mo_6_@GO material under photochemical conditions and, as a consequence, promote the decomposition of the catalyst [82]; and (ii) a loss of the stability of the coordinative bond between the {Mo_6_I^i^_8_}^4+^ cluster cores and the GO under catalytic conditions. The catalytic activity decrease in the cluster units in the presence of GO (vide supra) could be due, in part, to the low accessibility/activity of the Mo_6_I^i^_6_ cluster units covalently anchored onto GO surfaces [36], but mainly to the photodecomposition of the leached transient Mo_6_I^i^_6_ clusters without stabilizing carboxylate and alkoxo ligands.

In light of the above-mentioned results, and in order to prevent the decomposition of the cluster active sites, we decided to study the catalytic performance of the cluster compounds in the solid state, both as microcrystalline (TBA)_2_[Mo_6_I^i^_8_(O_2_CCH_3_)^a^_6_] and cluster-supported GO material, in the presence of water steam instead of liquid water. To the best of our knowledge, this is the first report of reactivity studies done with {Mo_6_X^i^_8_}^4+^ cluster core compounds in the presence of gas phase reactants.

### 3.4. Photocatalytic Activity of Microcrystalline (TBA)_2_[Mo_6_I^i^_8_(O_2_CCH_3_)^a^_6_] and (TBA)_2_Mo_6_I^i^_8_@GO in the Presence of Aqueous Mixtures in Vapor Phase

**Figure 7:**
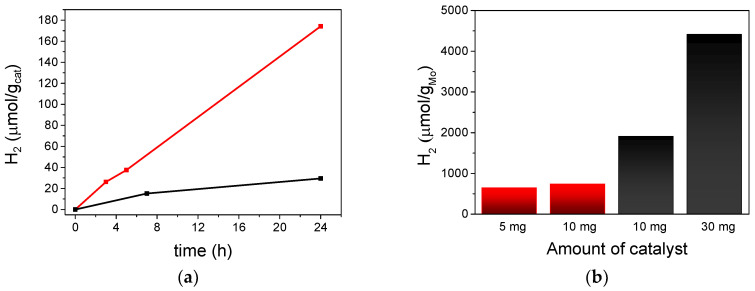
Amount of H_2_ evolved during the photocatalytic experiment in water/methanol in the vapor phase. (**a**) represents the reaction yields (μmol/g_cat_) of (TBA)_2_Mo_6_I^i^_8_@GO (black line) and (TBA)_2_[Mo_6_I^i^_8_(O_2_CCH_3_)^a^_6_] (red line) catalysts (10 mg); (**b**) represents the reaction yields (μmol/g_Mo_) of (TBA)_2_Mo_6_I^i^_8_@GO (in black) and (TBA)_2_[Mo_6_I^i^_8_(O_2_CCH_3_)^a^_6_] (in red) in different weights after 24 h of radiation.

Pure microcrystalline (TBA)_2_[Mo_6_I^i^_8_(O_2_CCH_3_)^a^_6_] compound was studied in the photocatalytic water reduction with methanol as the sacrificial reductant in the vapor phase. The amount of hydrogen generated relative to the molybdenum cluster content vs. time is plotted in Figure 7a. After long irradiation time, (TBA)_2_[Mo_6_I^i^_8_(O_2_CCH_3_)^a^_6_] provides 174 μmol/g_cat_ of H_2_. The turnover number (TON) calculated with respect to the cluster is 0.42, which is comparable to the value (0.34) reported for the Py_2_Mo compound [37]. Half the amount of the photocatalyst used gave similar yields (151 μmol/g_cat_). The yield obtained after 5 h of irradiation (Figure 7a) was about twenty times lower than the yield obtained in homogeneous conditions by using approximately the same amount of catalyst. This fact can be explained by considering the low specific surface area of the microcrystalline molecular material (3.6 m^2^/g) and, as a consequence, it has less cluster sites accessible to vapor water molecules if we compare it with the catalyst under homogeneous conditions.

The integrity of the catalyst was checked under the same reaction conditions. After it was recovered with dichloromethane, UV–Vis and ESI-MS analyses were performed. The absorption bands were the same after and before the reaction (Figure S14), and the mass analyses confirmed the integrity of the cluster complex after irradiation (Figure S15). These results indicate that the catalyst remained unaltered under photocatalytic water reduction conditions and for a long irradiation time.

In order to enhance and prolong the catalytic activity of the molybdenum cluster sites in the same gas phase conditions with respect to those available on the surface of the microcrystalline material, we switched to the highly dispersed cluster units grafted onto the graphenic surface of the (TBA)_2_Mo_6_I^i^_8_@GO nanomaterial, with the expectation that the catalytic performance of the cluster sites would improve. For these reasons, we were encouraged to study the catalytic performance of this nanomaterial in vapor phase.

**Figure 8:**
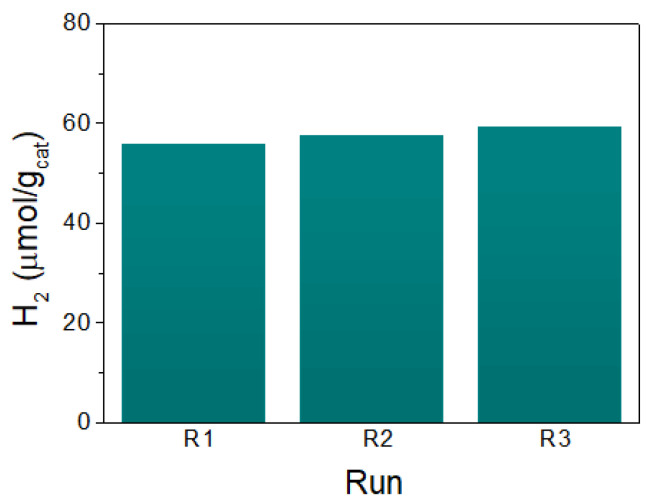
Recycling of the (TBA)_2_Mo_6_I^i^_8_@GO catalyst in the photochemical H_2_ production from water in vapor phase after 16 h of radiation.

Methanol was used as the sacrificial reductant under the same heterogeneous catalytic conditions as for the molecular precursor. The H_2_ production of (TBA)_2_Mo_6_I^i^_8_@GO was 30 μmol/g_cat_ (Figure 7a), and increased to 68 μmol/g_cat_ with threefold the catalyst amount. The hydrogen generation per atomic molybdenum was higher than the production achieved with the microcrystalline molecular compound (Figure 7b). We were thus able to demonstrate that the activity of the molybdenum cluster was enhanced in the hybrid catalyst. The TOF vs. atomic molybdenum was 2 × 10^−6^ s^−1^, which was threefold higher than the value obtained for the microcrystalline molecular precursor using the same amount of catalyst under gas phase conditions. This TOF was in the same order of magnitude as exhibited by the Py_2_Mo@Gene and (TBA)_2_Mo_6_Br^i^_8_@GO composites [36,37], and the H_2_ production rates were low, like those reported for other molybdenum/GO photocatalysts (Table S1). In addition, the recycling ability of the (TBA)_2_Mo_6_I^i^_8_@GO catalyst was examined after three consecutive reaction batches with 16 h of irradiation each, and the results are summarized in Figure 8. We observed that the recovered catalyst exhibited almost a similar yield up to three runs, demonstrating that the hybrid catalyst is highly recyclable without significant loss in activity.

**Figure 9:**
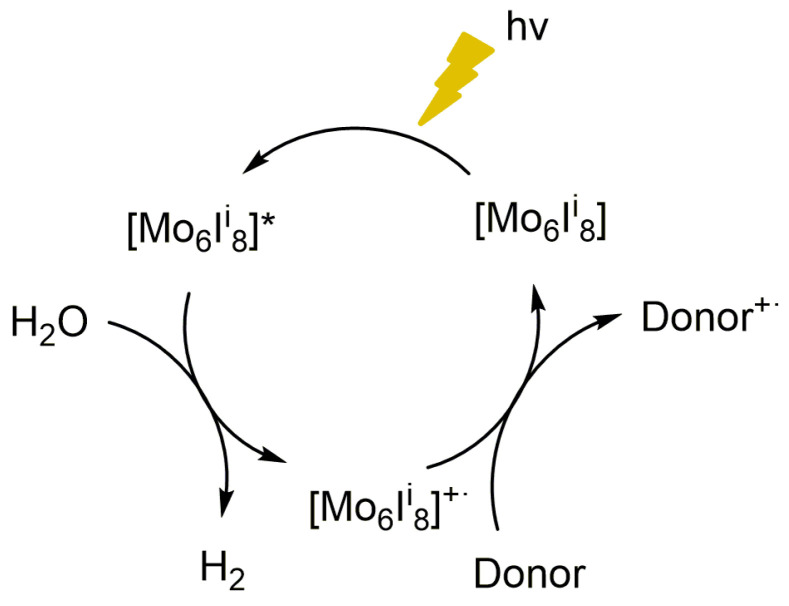
Plausible mechanism of photoreduction of vapor phase water to H_2_ catalyzed by the molybdenum cluster units of (TBA)_2_[Mo_6_I^i^_8_(O_2_CCH_3_)^a^_6_] and (TBA)_2_Mo_6_I^i^_8_@GO catalysts.

A possible mechanistic pathway of the catalytic HER is illustrated in Figure 9 on the basis of the low band gap values, good UV and visible light absorbance, and redox properties of the Mo_6_I^i^_8_ clusters. After the absorption of light, the {Mo_6_I^i^_8_}^4+^ cluster core was promoted to the excited state ([{Mo_6_I^i^_8_}^4+^]*) by HOMO to LUMO transition (Equation (1)). The singlet excited state (S_1_) of the {Mo_6_X_8_}^4+^ (X = Cl, Br, I) cluster core undergoes very efficient intersystem crossing to the triplet transition states (T_1_). We calculated the excited S_1_ energy from the absorption and electrochemical measurements and T_1_ energies from the emission spectrum for (TBA)_2_[Mo_6_I^i^_8_(O_2_CCH_3_)^a^_6_] (calculations and measurements are detailed in the last section of the Supplementary Materials and Figures S16–S17) to demonstrate that the {Mo_6_I^i^_8_}^4+^ cluster core is capable to transfer electrons to protons. Hence, the excited state transfers one electron per cluster unit to the water molecule and the two-electron transfer process would accomplish the generation of molecular hydrogen (Equations (2) and (3)). In the last step of the photocatalytic reaction, the positively charged cluster gets an electron from the sacrificial donor (TEA or MeOH) and the original cluster state is recovered (Equation (4)). This oxidative quenching mechanism is based in a two component system, in which the cluster acts simultaneously as a photosensitizer and as a catalyst for H_2_ production.

2[{Mo_6_I^i^_8_}^4+^] + h*ν* → 2[{Mo_6_I^i^_8_}^4+^]* (1)

2[{Mo_6_I^i^_8_}^4+^]* → 2[{Mo_6_I^i^_8_}^5+^]·+ 2e^−^ (2)

2H_2_O + 2e^−^ → H_2_ + 2OH^−^ (3)

2[{Mo_6_I^i^_8_}^5+^]·+ 2D → 2D^+^·+ 2[{Mo_6_I^i^_8_}^4+^] (4)

## 4. Conclusions

The catalytic activity and stability of the cluster complex (TBA)_2_[Mo_6_I^i^_8_(O_2_CCH_3_)^a^_6_] and the hybrid composite (TBA)_2_Mo_6_I^i^_8_@GO were investigated in the photoreduction of liquid and gas phase water under UV–Vis irradiation, and the best results were obtained in gas phase conditions. Novel (TBA)_2_Mo_6_I^i^_8_@GO material was prepared by coordinative anchoring of the cluster units, which implies the exchange reaction of the cluster-coordinated terminal acetate ligands with oxygen-containing functional groups on the GO surfaces, namely –OH and –COOH. The composition and morphological properties of this composite were determined by FTIR, Raman, UV–Vis, photoluminescence, XRD, HR-TEM, ICP-AES, and combustion elemental analysis.

The catalytic water photoreduction studies showed that: (i) liquid water reduction achieved the best hydrogen production yield (1326 μmol/g_cat_ of H_2_) by using cluster complex (TBA)_2_[Mo_6_I^i^_8_(O_2_CCH_3_)^a^_6_] dissolved in a water-organic mixture (50/45/5% v/v of H_2_O/acetone/TEA) after 5 h of irradiation, whereas the yield was significantly lower (291 μmol/g_cat_) when the hybrid composite (TBA)_2_Mo_6_I^i^_8_@GO was used under the same conditions. In both catalytic experiments, the catalysts degraded during the process: ESI-MS and single-crystal x-ray analyses revealed in situ generation of less active species (namely [Mo_6_I^i^_8_(OCOCH_3_)^a^_5_(OH)]^2−^, [Mo_6_I^i^_8_(OCOCH_3_)^a^_4_(OH)_2_]^2−^ (in solution), and [Mo_6_I^i^_8_(OH)^a^_4_(H_2_O)^a^_2_]·2H_2_O (as crystalline solid phase)) derived from the hydrolytic transformation of cluster complex (TBA)_2_[Mo_6_I^i^_8_(O_2_CCH_3_)^a^_6_]. The hybrid composite (TBA)_2_Mo_6_I^i^_8_@GO decomposed completely, and no synergetic effect between Mo_6_ units and GO was observed; (ii) water vapor reduction demonstrated for the first time a successful water vapor photocatalytic reduction using cluster complex (TBA)_2_[Mo_6_I^i^_8_(O_2_CCH_3_)^a^_6_] and the hybrid composite (TBA)_2_Mo_6_I^i^_8_@GO as catalysts. They remained intact and retained their catalytic activity for at least 24 h. The highest yield, obtained by using the microcrystalline (TBA)_2_[Mo_6_I^i^_8_(O_2_CCH_3_)^a^_6_], was 174 μmol/g_cat_, and this limited value is attributed to the smaller number of the cluster sites accessible to gas phase water molecules than in the catalyst used under homogeneous conditions, where each cluster unit can be accessed. The catalytic performance of the cluster sites was improved by using the (TBA)_2_Mo_6_I^i^_8_@GO composite as the catalyst under identical conditions. The TOF value with respect to the atomic content of molybdenum for (TBA)_2_Mo_6_I^i^_8_@GO (2 × 10^−6^ s^−1^) increased threefold over that for the microcrystalline (TBA)_2_[Mo_6_I^i^_8_(O_2_CCH_3_)^a^_6_]. The hybrid photocatalyst permits efficient recycling after at least three runs, and provided similar yields of H_2_ under identical experimental conditions.

This study demonstrates for the first time the possibility of using {Mo_6_I^i^_8_}^4+^ clusters (and hybrid materials based on the {Mo_6_I_8_}^4+^ clusters) in the catalytic process of water vapor reduction. The easy recovery, efficient recycling, and the robustness of both of the microcrystalline and the cluster-supported catalysts under vapor phase conditions make the developed methodology superior and more advantageous for photocatalytic production of molecular hydrogen from water.

## Supplementary Materials

The following are available online at https://www.mdpi.com/2079-4991/10/7/1259/s1, Figure S1: Photoreactor used for catalytic reactions using the microcrystalline catalyst and an aqueous solution in vapor phase, Figure S2: HER (μmol of H_2_/g_cat_) vs. time plot by using the (TBA)_2_[Mo_6_I^i^_8_(O_2_CCH_3_)^a^_6_] catalyst in the presence or water/TEA (30/5% v/v) mixtures with an organic co-solvent, namely: acetone (black line), DMF (red line), and acetonitrile (blue line), Figure S3: Representation of the reaction rates (μmol of H_2_/h·g_cat_) at 5 min (blue line) and 5 h (red line) by using the (TBA)_2_[Mo_6_I^i^_8_(O_2_CCH_3_)^a^_6_] catalyst in the presence of water/acetone/TEA mixture (50/45/5% v/v), Figure S4: Experimental (bottom) ESI mass generated molecular peaks of (from right to left): [Mo_6_I^i^_8_(O_2_CH_3_)^a^_6_]^2−^, [Mo_6_I^i^_8_(O_2_CH_3_)^a^_5_(OH)^a^]^2−^, and [Mo_6_I^i^_8_(O_2_CH_3_)^a^_4_(OH)^a^_2_]^2−^ detected of a reaction sample taken at 105 min reaction time in the catalytic photoreduction of water in liquid phase. Simulated (top) molecular peak for the [Mo_6_I^i^_8_(O_2_CH_3_)^a^_5_(OH)^a^]^2−^ species, Figure S5: Representation of [Mo_6_I^i^_8_(OH)^a^_4_(H_2_O)^a^_2_] according to single crystal x-ray structure determination. Displacement ellipsoids are shown at the 50% probability level, Figure S6: Projections of the [Mo_6_I^i^_8_(OH)^a^_4_(H_2_O)^a^_2_]·2H_2_O structure along the a, b, and c axis, Figure S7: Pairs of the solvate water molecules trapped between the clusters in the crystal packing of [Mo_6_I^i^_8_(OH)^a^_4_(H_2_O)^a^_2_]·2H_2_O. H bonding interactions between oxygen atoms are represented in dashed lines. Iodine atoms are omitted for clarity, Figure S8: ESI mass spectrum of a reaction sample taken after 24 h of illumination in the catalytic photoreduction of water in liquid phase, single-crystal structure determination, and refinement of [Mo_6_I^i^_8_(OH)^a^_4_(H_2_O)^a^_2_]·2H_2_O structural analysis, Figure S9: XRD patterns (from top to bottom) of (TBA)_2_[Mo_6_I^i^_8_(O_2_CCH_3_)^a^_6_], (TBA)_2_Mo_6_I^i^_8_@GO, and GO, Figure S10: Steady state photoluminescence spectra (λ_exc_ = 345 nm), which depicts the quenching of the (TBA)_2_[Mo_6_I^i^_8_(O_2_CCH_3_)^a^_6_] luminescence (initial concentration 10^−5^ M in DMF) upon the addition of increasing volumes of a stock dispersion of GO (2.5 mg/L) in DMF, Figure S11: Scanning transmission electron microscopy (STEM) image (a) and EDXA (b) of (TBA)_2_Mo_6_I^i^_8_@GO, Figure S12: HER (μmol of H_2_/g_cat_) vs. time plot by using the (TBA)_2_Mo_6_I^i^_8_@GO (11 mg, black line), GO (11 mg, blue line), and (TBA)_2_[Mo_6_I^i^_8_(O_2_CCH_3_)^a^_6_] (0.54 mg, red line) catalysts in aqueous solution containing water/acetone/TEA mixture (50/45/5% v/v), Figure S13: HER (μmol of H_2_/g_cat_) vs. time plot by using the (TBA)_2_Mo_6_I^i^_8_@GO (11 mg, black line) and the recycled solid (11 mg, orange line) in aqueous solution containing water/acetone/TEA mixture (50/45/5% v/v), Figure S14: UV–Vis spectra of the most characteristic bands of (TBA)_2_[Mo_6_I^i^_8_(O_2_CCH_3_)^a^_6_] before (black line) and after (orange line) catalytic reaction under vapor phase conditions, Figure S15: ESI mass generated molecular peaks (from right to left): [Mo_6_I^i^_8_(O_2_CH_3_)^a^_6_]^2−^, [Mo_6_I^i^_7_(O_2_CH_3_)^a^_5_Cl]^2−^, and [Mo_6_I^i^_8_(O_2_CH_3_)^a^_5_Br]^2−^ detected in a reaction sample after 24 h reaction time of the catalytic reaction in gas phase. The detection of two less intense peaks is ascribed to the presence of interferences in the electrospray source, calculation of the singlet and triplet excited states of the [Mo_6_I^i^_8_(O_2_CCH_3_)^a^_6_]^2−^ complex, Figure S16: Tauc plot of (TBA)_2_[Mo_6_I^i^_8_(O_2_CCH_3_)^a^_6_] from UV–Vis spectrum registered in acetonitrile, Figure S17: Emission spectrum of (TBA)_2_[Mo_6_I^i^_8_(O_2_CCH_3_)^a^_6_] acquired in acetonitrile, References, Table S1: Optimal catalytic activities of selected molybdenum and molybdenum-GO-based photocatalysts for H_2_ production from water.

## References

[1] Calise F., D’Accadia M.D., Santarelli M., Lanzini A., Ferrero D.B.T. (2019). Solar Hydrogen Production, Processes, Systems and Technologies.

[2] Ture E., Sheffield J.W., Sheffield Ç. (2007). Hydrogen Production from Solar Energy. Assessment of Hydrogen Energy for Sustainable Development.

[3] Dincer I., Zamfirescu C. (2017). Sustainable Hydrogen Production.

[4] Zou X., Zhang Y. (2015). Noble metal-free hydrogen evolution catalysts for water splitting. Chem. Soc. Rev..

[5] Koutavarapu R., Venkata Reddy C., Babu B., Reddy K.R., Cho M., Shim J. (2020). Carbon cloth/transition metals-based hybrids with controllable architectures for electrocatalytic hydrogen evolution—A review. Int. J. Hydrogen Energy.

[6] Babu B., Koutavarapu R., Shim J., Yoo K. (2020). Enhanced visible-light-driven photoelectrochemical and photocatalytic performance of Au-SnO_2_ quantum dot-anchored g-C_3_N_4_ nanosheets. Sep. Purif. Technol..

[7] Volonakis G., Giustino F. (2018). Surface properties of lead-free halide double perovskites: Possible visible-light photo-catalysts for water splitting. Appl. Phys. Lett..

[8] Yuan Y.-J., Chen D., Yu Z.-T., Zou Z.-G. (2018). Cadmium sulfide-based nanomaterials for photocatalytic hydrogen production. J. Mater. Chem. A.

[9] Cordier S., Grasset F., Molard Y., Amela-Cortes M., Boukherroub R., Ravaine S., Mortier M., Ohashi N., Saito N., Haneda H. (2015). Inorganic Molybdenum Octahedral Nanosized Cluster Units, Versatile Functional Building Block for Nanoarchitectonics. J. Inorg. Organomet. Polym. Mater..

[10] Nguyen N.T.K., Renaud A., Dierre B., Bouteille B., Wilmet M., Dubernet M., Ohashi N., Grasset F., Uchikoshi T. (2018). Extended Study on Electrophoretic Deposition Process of Inorganic Octahedral Metal Clusters: Advanced Multifunctional Transparent Nanocomposite Thin Films. Bull. Chem. Soc. Jpn..

[11] Nguyen T.K.N., Grasset F., Cordier S., Amela-Cortes M., Matsui Y., Ohashi N., Shirahata N., Uchikoshi T. (2020). Preparation and characterization of hollow silica nanocomposite functionalized with UV absorbable molybdenum cluster. Adv. Powder Technol..

[12] Renaud A., Nguyen T.K.N., Grasset F., Raissi M., Guillon V., Delabrouille F., Dumait N., Jouan P.-Y., Cario L., Jobic S. (2019). Preparation by electrophoretic deposition of molybdenum iodide cluster-based functional nanostructured photoelectrodes for solar cells. Electrochim. Acta.

[13] Daigre G., Cuny J., Lemoine P., Amela-Cortes M., Paofai S., Audebrand N., Le Gal La Salle A., Quarez E., Joubert O., Naumov N.G. (2018). Metal Atom Clusters as Building Blocks for Multifunctional Proton-Conducting Materials: Theoretical and Experimental Characterization. Inorg. Chem..

[14] Renaud A., Grasset F., Dierre B., Uchikoshi T., Ohashi N., Takei T., Planchat A., Cario L., Jobic S., Odobel F. (2016). Inorganic Molybdenum Clusters as Light-Harvester in All Inorganic Solar Cells: A Proof of Concept. ChemistrySelect.

[15] Vorotnikov Y.A., Efremova O.A., Vorotnikova N.A., Brylev K.A., Edeleva M.V., Tsygankova A.R., Smolentsev A.I., Kitamura N., Mironov Y.V., Shestopalov M.A. (2016). On the synthesis and characterisation of luminescent hybrid particles: Mo6 metal cluster complex/SiO_2_. RSC Adv..

[16] Nerambourg N., Aubert T., Neaime C., Cordier S., Mortier M., Patriarche G., Grasset F. (2014). Multifunctional hybrid silica nanoparticles based on [Mo_6_Br_14_]^2−^ phosphorescent nanosized clusters, magnetic γ-Fe_2_O_3_ and plasmonic gold nanoparticles. J. Colloid Interface Sci..

[17] Dechézelles J.-F., Aubert T., Grasset F., Cordier S., Barthou C., Schwob C., Maître A., Vallée R.A.L., Cramail H., Ravaine S. (2010). Fine tuning of emission through the engineering of colloidal crystals. Phys. Chem. Chem. Phys..

[18] Grasset F., Dorson F., Cordier S., Molard Y., Perrin C., Marie A.-M., Sasaki T., Haneda H., Bando Y., Mortier M. (2008). Water-in-Oil Microemulsion Preparation and Characterization of Cs_2_[Mo_6_X_14_]@SiO_2_ Phosphor Nanoparticles Based on Transition Metal Clusters (X = Cl, Br, and I). Adv. Mater..

[19] Robin M., Kuai W., Amela-Cortes M., Cordier S., Molard Y., Mohammed-Brahim T., Jacques E., Harnois M. (2015). Epoxy Based Ink as Versatile Material for Inkjet-Printed Devices. ACS Appl. Mater. Interfaces.

[20] Dybtsev D., Serre C., Schmitz B., Panella B., Hirscher M., Latroche M., Llewellyn P.L., Cordier S., Molard Y., Haouas M. (2010). Influence of [Mo_6_Br_8_F_6_]^2−^ Cluster Unit Inclusion within the Mesoporous Solid MIL-101 on Hydrogen Storage Performance. Langmuir.

[21] Vorotnikov Y.A., Pozmogova T.N., Solovieva A.O., Miroshnichenko S.M., Vorontsova E.V., Shestopalova L.V., Mironov Y.V., Shestopalov M.A., Efremova O.A. (2019). Luminescent silica mesoparticles for protein transduction. Mater. Sci. Eng. C.

[22] Elistratova J., Mukhametshina A., Kholin K., Nizameev I., Mikhailov M., Sokolov M., Khairullin R., Miftakhova R., Shammas G., Kadirov M. (2019). Interfacial uploading of luminescent hexamolybdenum cluster units onto amino-decorated silica nanoparticles as new design of nanomaterial for cellular imaging and photodynamic therapy. J. Colloid Interface Sci..

[23] Cheplakova A.M., Solovieva A.O., Pozmogova T.N., Vorotnikov Y.A., Brylev K.A., Vorotnikova N.A., Vorontsova E.V., Mironov Y.V., Poveshchenko A.F., Kovalenko K.A. (2017). Nanosized mesoporous metal–organic framework MIL-101 as a nanocarrier for photoactive hexamolybdenum cluster compounds. J. Inorg. Biochem..

[24] Neaime C., Amela-Cortes M., Grasset F., Molard Y., Cordier S., Dierre B., Mortier M., Takei T., Takahashi K., Haneda H. (2016). Time-gated luminescence bioimaging with new luminescent nanocolloids based on [Mo_6_I_8_(C_2_F_5_COO)_6_]^2−^ metal atom clusters. Phys. Chem. Chem. Phys..

[25] Solovieva A.O., Vorotnikov Y.A., Trifonova K.E., Efremova O.A., Krasilnikova A.A., Brylev K.A., Vorontsova E.V., Avrorov P.A., Shestopalova L.V., Poveshchenko A.F. (2016). Cellular internalisation, bioimaging and dark and photodynamic cytotoxicity of silica nanoparticles doped by {Mo_6_I_8_}^4+^ metal clusters. J. Mater. Chem. B.

[26] Aubert T., Cabello-Hurtado F., Esnault M.-A., Neaime C., Lebret-Chauvel D., Jeanne S., Pellen P., Roiland C., Le Polles L., Saito N. (2013). Extended Investigations on Luminescent Cs_2_[Mo_6_Br_14_]@SiO_2_ Nanoparticles: Physico-Structural Characterizations and Toxicity Studies. J. Phys. Chem. C.

[27] Kirakci K., Kubát P., Fejfarová K., Martinčík J., Nikl M., Lang K. (2016). X-ray Inducible Luminescence and Singlet Oxygen Sensitization by an Octahedral Molybdenum Cluster Compound: A New Class of Nanoscintillators. Inorg. Chem..

[28] Evtushok D.V., Melnikov A.R., Vorotnikova N.A., Vorotnikov Y.A., Ryadun A.A., Kuratieva N.V., Kozyr K.V., Obedinskaya N.R., Kretov E.I., Novozhilov I.N. (2017). A comparative study of optical properties and X-ray induced luminescence of octahedral molybdenum and tungsten cluster complexes. Dalt. Trans..

[29] Kirakci K., Zelenka J., Rumlová M., Cvačka J., Ruml T., Lang K. (2019). Cationic octahedral molybdenum cluster complexes functionalized with mitochondria-targeting ligands: Photodynamic anticancer and antibacterial activities. Biomater. Sci..

[30] Nagashima S., Kamiguchi S., Chihara T. (2014). Catalytic Reactions over Halide Cluster Complexes of Group 5–7 Metals. Metals.

[31] Kamiguchi S., Nagashima S., Chihara T. (2014). Characterization of Catalytically Active Octahedral Metal Halide Cluster Complexes. Metals.

[32] Barras A., Cordier S., Boukherroub R. (2012). Fast photocatalytic degradation of rhodamine B over [Mo_6_Br_8_(N_3_)_6_]^2−^ cluster units under sun light irradiation. Appl. Catal. B Environ..

[33] Barras A., Das M.R., Devarapalli R.R., Shelke M.V., Cordier S., Szunerits S., Boukherroub R. (2013). One-pot synthesis of gold nanoparticle/molybdenum cluster/graphene oxide nanocomposite and its photocatalytic activity. Appl. Catal. B Environ..

[34] Kumar P., Mungse H.P., Cordier S., Boukherroub R., Khatri O.P., Jain S.L. (2015). Hexamolybdenum clusters supported on graphene oxide: Visible-light induced photocatalytic reduction of carbon dioxide into methanol. Carbon.

[35] Beltrán A., Mikhailov M., Sokolov M.N., Pérez-Laguna V., Rezusta A., Revillo M.J., Galindo F. (2016). A photobleaching resistant polymer supported hexanuclear molybdenum iodide cluster for photocatalytic oxygenations and photodynamic inactivation of Staphylococcus aureus. J. Mater. Chem. B.

[36] Feliz M., Puche M., Atienzar P., Concepción P., Cordier S., Molard Y. (2016). In Situ Generation of Active Molybdenum Octahedral Clusters for Photocatalytic Hydrogen Production from Water. ChemSusChem.

[37] Feliz M., Atienzar P., Amela-Cortés M., Dumait N., Lemoine P., Molard Y., Cordier S. (2019). Supramolecular Anchoring of Octahedral Molybdenum Clusters onto Graphene and Their Synergies in Photocatalytic Water Reduction. Inorg. Chem..

[38] Ivanova M.N., Vorotnikov Y.A., Plotnikova E.E., Marchuk M.V., Ivanov A.A., Asanov I.P., Tsygankova A.R., Grayfer E.D., Fedorov V.E., Shestopalov M.A. (2020). Hexamolybdenum Clusters Supported on Exfoliated h-BN Nanosheets for Photocatalytic Water Purification. Inorg. Chem..

[39] Kumar P., Kumar S., Cordier S., Paofai S., Boukherroub R., Jain S.L. (2014). Photoreduction of CO2 to methanol with hexanuclear molybdenum [Mo_6_Br_14_]^2−^ cluster units under visible light irradiation. RSC Adv..

[40] Prévôt M., Amela-Cortes M., Manna S.K., Lefort R., Cordier S., Folliot H., Dupont L., Molard Y. (2015). Design and Integration in Electro-Optic Devices of Highly Efficient and Robust Red-NIR Phosphorescent Nematic Hybrid Liquid Crystals Containing [Mo_6_I_8_(OCOC_n_F_2n+1_)_6_]^2−^ (n = 1, 2, 3) Nanoclusters. Adv. Funct. Mater..

[41] Sokolov M.N., Mihailov M.A., Peresypkina E.V., Brylev K.A., Kitamura N., Fedin V.P. (2011). Highly luminescent complexes [Mo_6_X_8_(n-C_3_F_7_COO)_6_]^2−^ (X = Br, I). Dalt. Trans..

[42] Kirakci K., Kubát P., Dušek M., Fejfarová K., Šícha V., Mosinger J., Lang K. (2012). A Highly Luminescent Hexanuclear Molybdenum Cluster—A Promising Candidate toward Photoactive Materials. Eur. J. Inorg. Chem..

[43] Kirakci K., Kubat P., Langmaier J., Polivka T., Fuciman M., Fejfarova K., Lang K. (2013). A comparative study of the redox and excited state properties of (nBu_4_N)_2_[Mo_6_X_14_] and (nBu_4_N)_2_[Mo_6_X_8_(CF_3_COO)_6_] (X = Cl, Br, or I). Dalt. Trans..

[44] Efremova O.A., Shestopalov M.A., Chirtsova N.A., Smolentsev A.I., Mironov Y.V., Kitamura N., Brylev K.A., Sutherland A.J. (2014). A highly emissive inorganic hexamolybdenum cluster complex as a handy precursor for the preparation of new luminescent materials. Dalt. Trans..

[45] Efremova O.A., Vorotnikov Y.A., Brylev K.A., Vorotnikova N.A., Novozhilov I.N., Kuratieva N.V., Edeleva M.V., Benoit D.M., Kitamura N., Mironov Y.V. (2016). Octahedral molybdenum cluster complexes with aromatic sulfonate ligands. Dalt. Trans..

[46] Mikhailov M.A., Brylev K.A., Abramov P.A., Sakuda E., Akagi S., Ito A., Kitamura N., Sokolov M.N. (2016). Synthetic Tuning of Redox, Spectroscopic, and Photophysical Properties of {Mo_6_I_8_}^4+^ Core Cluster Complexes by Terminal Carboxylate Ligands. Inorg. Chem..

[47] Riehl L., Seyboldt A., Ströbele M., Enseling D., Jüstel T., Westberg M., Ogilby P.R., Meyer H.J. (2016). A ligand substituted tungsten iodide cluster: Luminescence vs. singlet oxygen production. Dalt. Trans..

[48] Sokolov M.N., Brylev K.A., Abramov P.A., Gallyamov M.R., Novozhilov I.N., Kitamura N., Mikhaylov M.A. (2017). Complexes of {W6I8}4+ Clusters with Carboxylates: Preparation, Electrochemistry, and Luminescence. Eur. J. Inorg. Chem..

[49] Mikhaylov M.A., Sokolov M.N. (2019). Molybdenum Iodides–from Obscurity to Bright Luminescence. Eur. J. Inorg. Chem..

[50] Yam K.M., Guo N., Jiang Z., Li S., Zhang C. (2020). Graphene-Based Heterogeneous Catalysis: Role of Graphene. Catalysts.

[51] Huang C., Li C., Shi G. (2012). Graphene based catalysts. Energy Environ. Sci..

[52] Xiang Q., Yu J., Jaroniec M. (2012). Graphene-based semiconductor photocatalysts. Chem. Soc. Rev..

[53] Yeh T.-F., Syu J.-M., Cheng C., Chang T.-H., Teng H. (2010). Graphite Oxide as a Photocatalyst for Hydrogen Production from Water. Adv. Funct. Mater..

[54] Yeh T.-F., Cihlář J., Chang C.-Y., Cheng C., Teng H. (2013). Roles of graphene oxide in photocatalytic water splitting. Mater. Today.

[55] Latorre-Sánchez M., Lavorato C., Puche M., Fornés V., Molinari R., Garcia H. (2012). Visible-Light Photocatalytic Hydrogen Generation by Using Dye-Sensitized Graphene Oxide as a Photocatalyst. Chem. A Eur. J..

[56] Lerf A., He H., Riedl T., Forster M., Klinowski J. (1997). 13C and 1H MAS NMR studies of graphite oxide and its chemically modified derivatives. Solid State Ion..

[57] Konios D., Stylianakis M.M., Stratakis E., Kymakis E. (2014). Dispersion behaviour of graphene oxide and reduced graphene oxide. J. Colloid Interface Sci..

[58] Kharisov B.I., Kharissova O.V., Vázquez Dimas A., Gómez De La Fuente I., Peña Méndez Y. (2016). Review: Graphene-supported coordination complexes and organometallics: Properties and applications. J. Coord. Chem..

[59] Axet M.R., Dechy-Cabaret O., Durand J., Gouygou M., Serp P. (2016). Coordination chemistry on carbon surfaces. Coord. Chem. Rev..

[60] Axet M.R., Durand J., Gouygou M., Serp P. (2019). Surface coordination chemistry on graphene and two-dimensional carbon materials for well-defined single atom supported catalysts. Adv. Organomet. Chem..

[61] Arora S., Gupta N., Singh V. (2020). Improved Pd/Ru metal supported graphene oxide nano-catalysts for hydrodeoxygenation (HDO) of vanillyl alcohol, vanillin and lignin. Green Chem..

[62] Zhu S., Wang J., Fan W. (2015). Graphene-based catalysis for biomass conversion. Catal. Sci. Technol..

[63] Das V.K., Shifrina Z.B., Bronstein L.M. (2017). Graphene and graphene-like materials in biomass conversion: Paving the way to the future. J. Mater. Chem. A.

[64] Kumar S., Khatri O.P., Cordier S., Boukherroub R., Jain S.L. (2015). Graphene Oxide Supported Molybdenum Cluster: First Heterogenized Homogeneous Catalyst for the Synthesis of Dimethylcarbonate from CO_2_ and Methanol. Chem. A Eur. J..

[65] Sheldon J.C. (1962). 76. Bromo- and iodo-molybdenum(II) compounds. J. Chem. Soc..

[66] Schreck M., Niederberger M. (2019). Photocatalytic Gas Phase Reactions. Chem. Mater..

[67] Lapicque F., Lédé J., Villermaux J. (1986). Design and optimization of a reactor for high temperature dissociation of water and carbon dioxide using solar energy. Chem. Eng. Sci..

[68] Dionigi F., Vesborg P.C.K., Pedersen T., Hansen O., Dahl S., Xiong A., Maeda K., Domen K., Chorkendorff I. (2011). Gas phase photocatalytic water splitting with Rh_2__−y_CryO_3_/GaN:ZnO in μ-reactors. Energy Environ. Sci..

[69] Volostnykh M.V., Mikhaylov M.A., Sinelshchikova A.A., Kirakosyan G.A., Martynov A.G., Grigoriev M.S., Piryazev D.A., Tsivadze A.Y., Sokolov M.N., Gorbunova Y.G. (2019). Hybrid organic–inorganic supramolecular systems based on a pyridine end-decorated molybdenum(ii) halide cluster and zinc(ii) porphyrinate. Dalt. Trans..

[70] Felip-León C., Puche M., Miravet J.F., Galindo F., Feliz M. (2019). A spectroscopic study to assess the photogeneration of singlet oxygen by graphene oxide. Mater. Lett..

[71] Marcano D.C., Kosynkin D.V., Berlin J.M., Sinitskii A., Sun Z., Slesarev A., Alemany L.B., Lu W., Tour J.M. (2010). Improved Synthesis of Graphene Oxide. ACS Nano.

[72] (2019). CrysAlisPro Agilent Technologies.

[73] Puche M. (2017). Nanomateriales Híbridos Basados en Complejos de Metales de Transición Anclados Sobre óxido de Grafeno. Ph.D. Thesis.

[74] Montes-Navajas P., Asenjo N.G., Santamaría R., Menéndez R., Corma A., García H. (2013). Surface Area Measurement of Graphene Oxide in Aqueous Solutions. Langmuir.

[75] Povedailo V.A., Ronishenko B.V., Stepuro V.I., Tsybulsky D.A., Shmanai V.V., Yakovlev D.L. (2018). Fluorescence Quenching of Dyes by Graphene Oxide. J. Appl. Spectrosc..

[76] Liu Y., Liu C., Liu Y. (2011). Investigation on fluorescence quenching of dyes by graphite oxide and graphene. Appl. Surf. Sci..

[77] De Miguel M., Álvaro M., García H. (2012). Graphene as a Quencher of Electronic Excited States of Photochemical Probes. Langmuir.

[78] Nannelli P., Block B.P. (1968). Molybdenum(II) cluster compounds involving alkoxy groups. Inorg. Chem..

[79] Brničević N., Bašic I., Hoxha B., Planinić P., McCarley R.E. (2003). Molybdenum and tungsten methoxo clusters with differently bonded methoxo groups: Crystal structure of [Na(CH_3_OH)_5_]_2_[Mo_6_(μ_3_-Br)_8_(OCH_3_)_6_]. Polyhedron.

[80] Schoonover J.R., Zietlow T.C., Clark D.L., Heppert J.A., Chisholm M.H., Gray H.B., Sattelberger A.P., Woodruff W.H. (1996). Resonance Raman Spectra of [M_6_X_8_Y_6_]^2−^ Cluster Complexes (M = Mo, W; X, Y = Cl, Br, I). Inorg. Chem..

[81] Gao W., Alemany L.B., Ci L., Ajayan P.M. (2009). New insights into the structure and reduction of graphite oxide. Nat. Chem..

[82] Gurunathan S., Woong Han J., Kim J. (2013). Green chemistry approach for the synthesis of biocompatible graphene. Int. J. Nanomed..

