# Supplementary material for: Enhanced Photocatalytic Activity and Stability in Hydrogen Evolution of Mo6 Iodide Clusters Supported on Graphene Oxide
Source: Nanomaterials (Basel). 2020 Jun 28;10(7):1259. doi: 10.3390/nano10071259 (PMC7407389; doi:10.3390/nano10071259)
Supplement: Supplementary file 1 [file nanomaterials-10-01259-s001.pdf]

Supplementary Materials

Enhanced Photocatalytic Activity and Stability in Hydrogen Evolution of Mo_6 Iodide Clusters Supported on Graphene Oxide

Marta Puche [1], Rocío García-Aboal [1], Maxim A. Mikhaylov [2], Maxim N. Sokolov [2], Pedro Atienzar [1] and Marta Feliz [1,*]

[1] Instituto de Tecnología Química, Universitat Politècnica de València-Consejo Superior de Investigaciones Científicas, Avenida de los Naranjos s/n, 46022 Valencia, Spain; mpuche@itq.upv.es (M.P.); rogarab@itq.upv.es (R.G.-A.); pedatcor@itq.upv.es (P.A.)

[2] Nikolaev Institute of Inorganic Chemistry, Siberian Branch of the Russian Academy of Sciences, 3 Acad. Lavrentiev Ave., Novosibirsk, 630090, Russia; mikhajlovmaks@yandex.ru (M.A.M.); caesar@niic.nsc.ru (M.N.S.)

* Correspondence: mfeliz@itq.upv.es; Tel.: +34-96-387-9696

TABLE OF CONTENTS

Figure S1. Photoreactor used for catalytic reactions using the microcrystalline catalyst and an aqueous solution in vapor phase.

Figure S2. HER (μmol of H_2/g_{cat}) *vs* time plot by using the $(TBA)_2[Mo_6I^i_8(O_2CCH_3)^a_6]$ catalyst in the presence or water/TEA (30/5% v/v) mixtures with an organic co-solvent, namely: acetone (black line), DMF (red line) and acetonitrile (blue line).

Table S1. Optimal catalytic activities of selected molybdenum and molybdenum-GO-based photocatalysts for H_2 production from water.

Figure S3. Representation of the reaction rates (μmol of $H_2/h{\cdot}g_{cat}$) at 5 min (blue line) and 5 h (red line) by using the $(TBA)_2[Mo_6I^i_8(O_2CCH_3)^a_6]$ catalyst in the presence of water/acetone/TEA mixture (50/45/5% v/v).

Figure S4. Experimental (bottom) ESI mass generated molecular peaks of (from right to left): $[Mo_6I^i_8(O_2CH_3)^a_6]^{2-}$, $[Mo_6I^i_8(O_2CH_3)^a_5(OH)^a]^{2-}$ and $[Mo_6I^i_8(O_2CH_3)^a_4(OH)^a_2]^{2-}$ detected of a reaction sample taken at 105 min reaction time in the catalytic photoreduction of water in liquid phase. Simulated (top) molecular peak for the $[Mo_6I^i_8(O_2CH_3)^a_5(OH)^a]^{2-}$ species.

Single-crystal structure determination and refinement of $[Mo_6I^i_8(OH)^a_4(H_2O)^a_2]{\cdot}2H_2O$. Structural analysis.

Figure S5. Representation of $[Mo_6I^i_8(OH)^a_4(H_2O)^a_2]$ according to single crystal X-ray structure determinantion. Displacement ellipsoids are shown at the 50% probability level.

Figure S6. Projections of the $[Mo_6I^i_8(OH)^a_4(H_2O)^a_2]{\cdot}2H_2O$ structure along the a, b and c axis.

Figure S7. Pairs of the solvate water molecules trapped between the clusters in the crystal packing of $[Mo_6I^i_8(OH)^a_4(H_2O)^a_2]{\cdot}2H_2O$. H bonding interactions between oxygen atoms are represented in dashed lines. Iodine atoms are omitted for clarity.

Figure S8. ESI mass spectrum of a reaction sample taken after 24 h of illumination in the catalytic photoreduction of water in liquid phase.

Figure S9. XRD patterns (from top to bottom) of $(TBA)_2[Mo_6I^i_8(O_2CCH_3)^a_6]$, $(TBA)_2Mo_6I^i_8$@GO and GO.

Figure S10. Steady state photoluminescence spectra (λ_{exc} = 345 nm) which depict the quenching of the $(TBA)_2[Mo_6I^i_8(O_2CCH_3)^a_6]$ luminescence (initial concentration 10^{-5} M in DMF) upon addition of increasing volumes of a stock dispersion of GO (2.5 mg/L) in DMF.

Figure S11. Scanning transmission electron microscopy (STEM) image (a) and EDXA (b) of $(TBA)_2Mo_6I^i_8$@GO.

Figure S12. HER (μmol of H_2/g_{cat}) *vs* time plot by using the $(TBA)_2Mo_6I^i_8$@GO (11 mg, black line), GO (11 mg, blue line) and $(TBA)_2[Mo_6I^i_8(O_2CCH_3)^a_6]$ (0.54 mg, red line) catalysts in aqueous solution containing water/acetone/TEA mixture (50/45/5% v/v).

Figure S13. HER (μmol of H_2/g_{cat}) *vs* time plot by using the $(TBA)_2Mo_6I^i_8$@GO (11 mg, black line) and the recycled solid (11 mg, orange line) in aqueous solution containing water/acetone/TEA mixture (50/45/5% v/v).

Figure S14. UV-vis spectra of the most characteristic bands of $(TBA)_2[Mo_6I^i_8(O_2CCH_3)^a_6]$ before (black line) and after (orange line) catalytic reaction under vapor phase conditions.

Figure S15. ESI mass generated molecular peaks (from right to left): $[Mo_6I^i_8(O_2CH_3)^a_6]^{2-}$, $[Mo_6I^i_7(O_2CH_3)^a_5Cl]^{2-}$ and $[Mo_6I^i_8(O_2CH_3)^a_5Br]^{2-}$ detected of a reaction sample after 24 h reaction time of the catalytic reaction in gas phase. The detection of two less intense peaks is ascribed to the presence of interferences in the electrospray source.

Calculation of the singlet and triplet excited states of the $[Mo_6I^i_8(O_2CCH_3)^a_6]^{2-}$ complex.

Figure S16. Tauc plot of $(TBA)_2[Mo_6I^i_8(O_2CCH_3)^a_6]$ from UV-vis spectrum registered in acetonitrile.

Figure S17. Emission spectrum of $(TBA)_2[Mo_6I^i_8(O_2CCH_3)^a_6]$ acquired in acetonitrile.

References

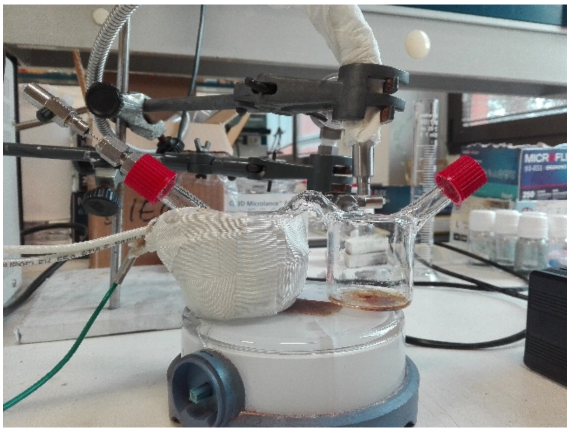

Figure S1. Photoreactor used for catalytic reactions using the microcrystalline catalyst and an aqueous solution in vapor phase.

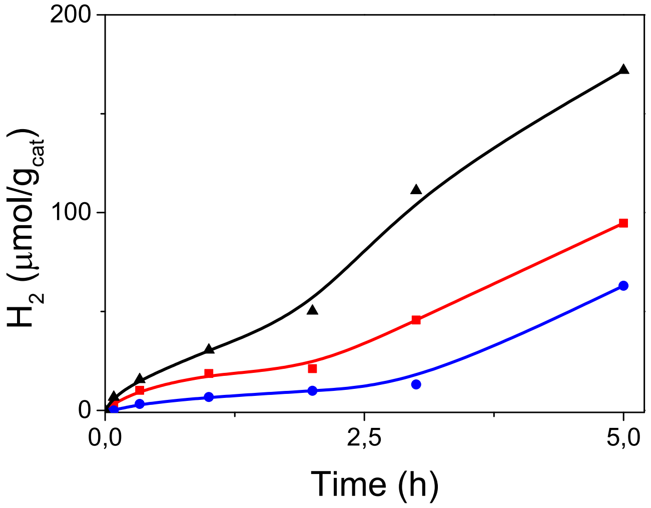

Figure S2. HER (µmol of H_2/g_{cat}) *vs* time plot by using the $(TBA)_2[Mo_6I^i_8(O_2CCH_3)^a_6]$ catalyst in the presence or water/TEA (30/5% v/v) mixtures with an organic co-solvent, namely: acetone (black line), DMF (red line) and acetonitrile (blue line).

Table S1. Optimal catalytic activities of selected molybdenum and molybdenum-GO-based photocatalysts for H_2 production from water. [a]

Photocatalyst	Sacrificial agent	Co-catalyst /Photosensitizer	Activity (H_2 production, $\mu mol \cdot g_{cat}^{-1} \cdot h^{-1}$)	Ref.
**$(TBA)_2[Mo_6I^i_8(O_2CCH_3)^a_6]$**	TEA	None	265 [c]	This work
**$(TBA)_2[Mo_6I^i_8(O_2CCH_3)^a_6]$**	MeOH	None	7 [d]	This work
$(TBA)([Mo_3S_7Br_4\{(CO_2Me_2)bpy\}Br])$	Na_2S/Na_2SO_3	None	6 [c]	[1]
MoS_2 (nanosheets)	Na_2S/ Na_2SO_3	None	47 [c]	[2]
MoS_2 nanoparticles (amorphous)	ascorbic acid	$[Ru(bpy)_3]^{2+}$	210 [c]	[3]
**$(TBA)_2Mo_6I^i_8$@GO**	MeOH	None	3 [d]	This work
$(TBA)_2Mo_6Br^i_8$@GO	MeOH	None	2 [c]	[4]
MoS_2 (bulk)/GO	Na_2S	None	28 [c]	[5]
MoS_2 (NFs)/GO	Na_2S	None	105 [c]	[5]
MoS_2 (NPs)/GO	Na_2S	None	221 [c]	[5]
MoS_2/SiC/GO [b]	Na_2S/ Na_2SO_3	MoS_2	66 [c]	[6]
MoS_2/SiC/GO [b]	Na_2S/ Na_2SO_3	MoS_2	87 [c]	[7]

[a] Abbreviations: bpy = 2,2′-bipyridine; NFs = nanoflowers; NPs = nanoparticles; BP = Black phosphorous; [b] 8 wt% Mo content for these hybrids; [c] Results obtained from aqueous mixtures in liquid phase; [d] Results obtained from aqueous mixtures in vapor phase.

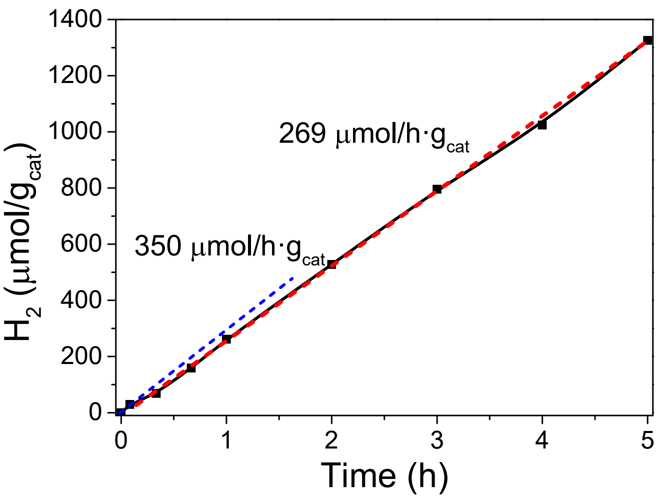

Figure S3. Representation of the reaction rates (µmol of H_2/h·g_{cat}) at 5 min (blue line) and 5 h (red line) by using the $(TBA)_2[Mo_6I^i_8(O_2CCH_3)^a_6]$ catalyst in the presence of water/acetone/TEA mixture (50/45/5% v/v).

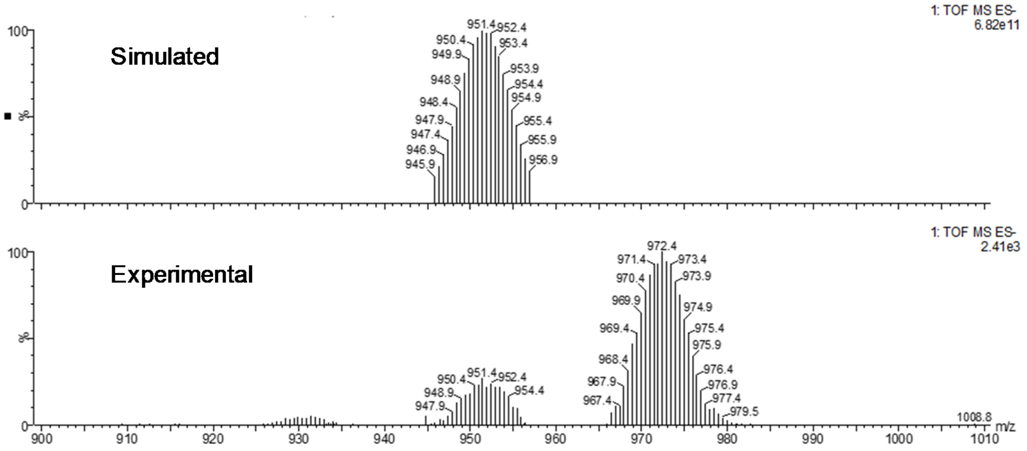

Figure S4. Experimental (bottom) ESI mass generated molecular peaks of (from right to left): $[Mo_6I^i_8(O_2CH_3)^a_6]^{2-}$, $[Mo_6I^i_8(O_2CH_3)^a_5(OH)^a]^{2-}$ and $[Mo_6I^i_8(O_2CH_3)^a_4(OH)^a_2]^{2-}$ detected of a reaction sample taken at 105 min reaction time in the catalytic photoreduction of water in liquid phase. Simulated (top) molecular peak for the $[Mo_6I^i_8(O_2CH_3)^a_5(OH)^a]^{2-}$ species.

Single-crystal structure determination and refinement of $[Mo_6I^i_8(OH)^a_4(H_2O)^a_2]\cdot 2H_2O$. Structural analysis.

The structure was solved using direct methods in SHELXS-2018/3 and refined by the full-matrix method based on F^2 with the program SHELXL-2014/7 with the aid of the OLEX2 (vs 1.2.10) [8–10]. Crystal size: 0.091 × 0.086 × 0.062 mm^3. Crystal data for $[Mo_6I^i_8(OH)^a_4(H_2O)^a_2]\cdot 2H_2O$: $O_8I_8Mo_6$, M = 1718.81, trigonal, *R*-3*c* space group, a = b = 9.6019(2) Å, c = 43.4031(12) Å, $\alpha = \beta = 90.0$ °, $\gamma = 120.0$ ° V = 3465.50(17) $Å^3$, T = 200.00(14) K, Z = 6, μ = 110.051 mm^{-1}, ρ_{calc} = 4.942 $g.cm^{-3}$, θ range for data collection: 11.396 - 133.094 °, 9809 reflection collected, 691 unique reflections, R_{int} = 0.0406, completeness to theta max = 1.000. Final refinement converged with R_1 = 0.0231 and wR_2 = 0.0610 for all reflections, GOF = 1.133, max/min residual electron density 0.33/-0.25 e·$Å^{-3}$. The detailed crystal description and representations of the structure are included in the Supporting Information section. Further details of the crystal structure investigation(s) may be obtained from the Fachinformationszentrum Karlsruhe, 76344 Eggenstein-Leopoldshafen, Germany (fax: (+49)7247-808-666; e-mail: crysdata@fiz-karlsruhe.de), on quoting the deposition number CCDC-1998094.

The $[Mo_6I^i_8(OH)^a_4(H_2O)^a_2]\cdot 2H_2O$ compound crystallizes in the *R*-3*c* space group. Anisotropic displacement parameters were refined for all atoms of the structure. The cluster unit is based on a $\{Mo_6I^i_8\}^{4+}$ cluster core, with 12 x Mo-Mo = 2.633 Å, 3 × Mo-I = 2.793 Å and 3 × Mo-I = 2.801 Å interatomic distances, which is additionally bonded to six oxygen-donor ligands in apical positions. The quality of the data did not allow discriminating the position of hydrogen atoms, and the hydrogen atoms were not included in calculated positions riding on the respective oxygen atoms. Since all Mo-O1 distances (2.120(4) Å) are equivalent, a statistical disorder of four OH^- and two H_2O ligands has been assigned, by analogy to the reported structure with the same formula, which resulted isostructural, with Mo-O1 interatomic distances of 2.125(4) Å [11]. This reported compound was also obtained from alkaline aqueous solutions, but as the product of hydrolysis of $[Mo_6I_{14}]^{2-}$ at pH = 9.18 from a buffer 0.1 M $Na_2B_4O_7$ solution.

The $[Mo_6I^i_8(OH)^a_4(H_2O)^a_2]$ units (Figure S5) are arranged according to a hcp stacking (Figure S6) and the cohesion of the structure is based on a hydrogen bonding network (Figure S7).The octahedral cavities are filled with discrete aggregates formed by $(H_2O)_2$ dimers with O2···O2 distances of 2.776 Å. These aggregates are embedded into a ''matrix'' of six O1 atoms from six different clusters, in which each O1 interacts not only with one another O1 but also to one O2 by hydrogen bonds, with O1···O2 and O1···O1 distances of 2.774 and 2.643 Å, respectively. The non-coordinated water molecules participate in four hydrogen bonds, and the terminal H_2O and OH^- ligands participate in two hydrogen bonds.

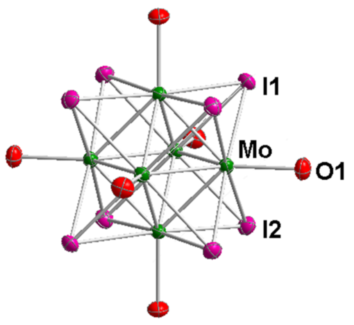

Figure S5. Representation of $[Mo_6I^i_8(OH)^a_4(H_2O)^a_2]$ according to single crystal X-ray structure determinantion. Displacement ellipsoids are shown at the 50% probability level.

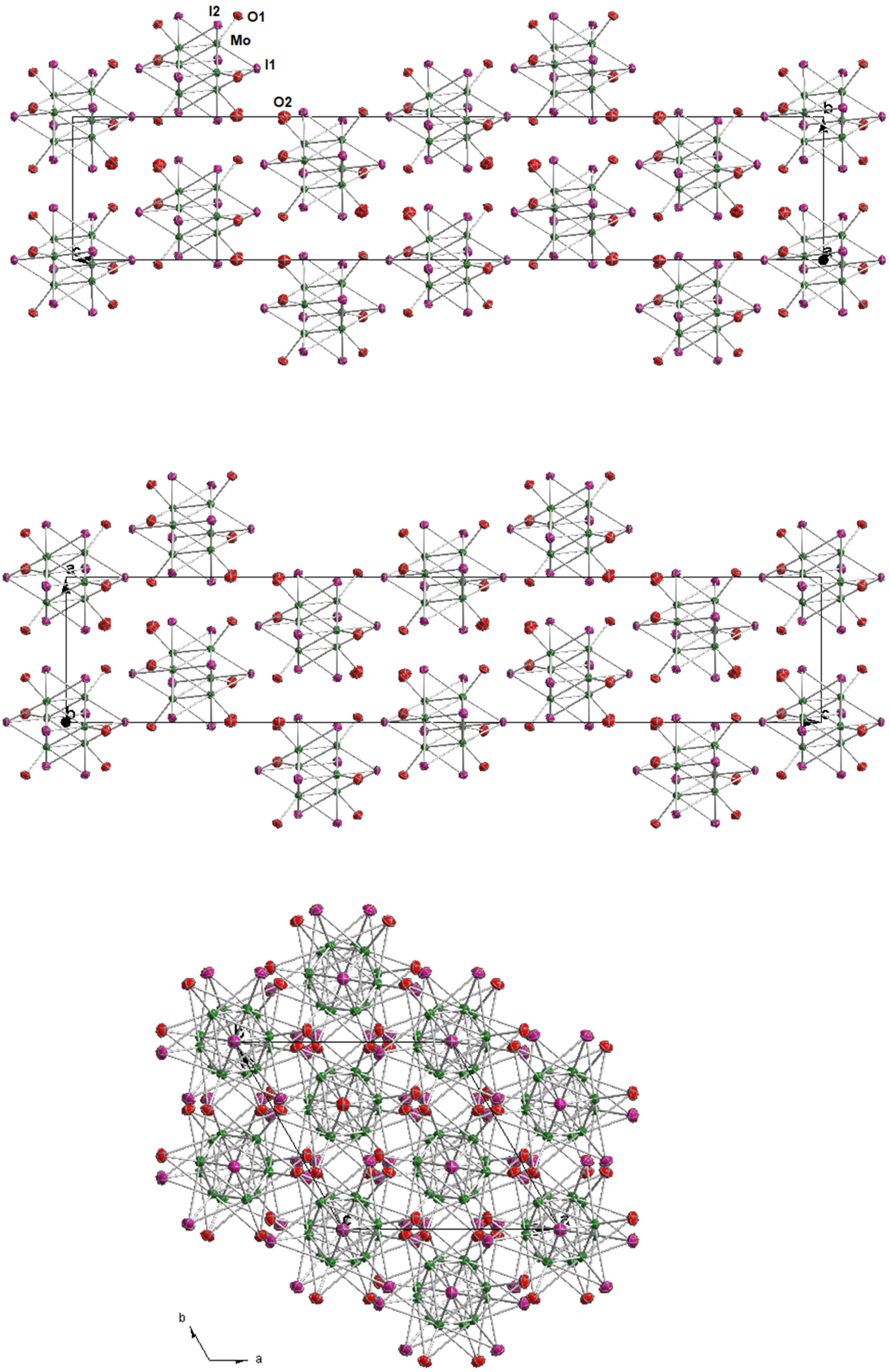

Figure S6. Projections of the $[Mo_6I^i_8(OH)^a_4(H_2O)^a_2]\cdot 2H_2O$ structure along the a, b and c axis.

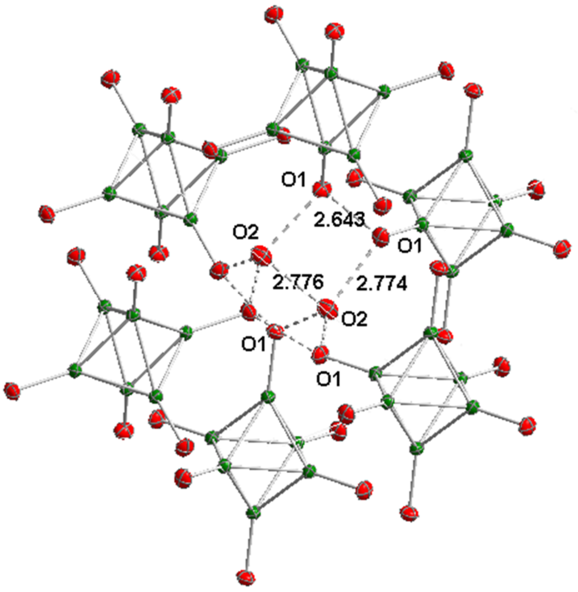

Figure S7. Pairs of the solvate water molecules trapped between the clusters in the crystal packing of $[Mo_6I^i_8(OH)^a_4(H_2O)^a_2]\cdot 2H_2O$. H bonding interactions between oxygen atoms are represented in dashed lines. Iodine atoms are omitted for clarity.

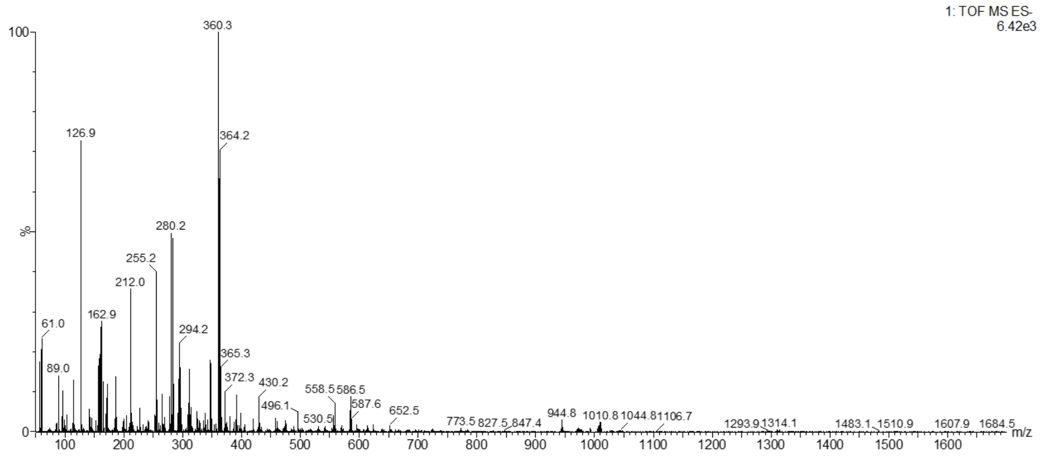

Figure S8. ESI mass spectrum of a reaction sample taken after 24 h of illumination in the catalytic photoreduction of water in liquid phase.

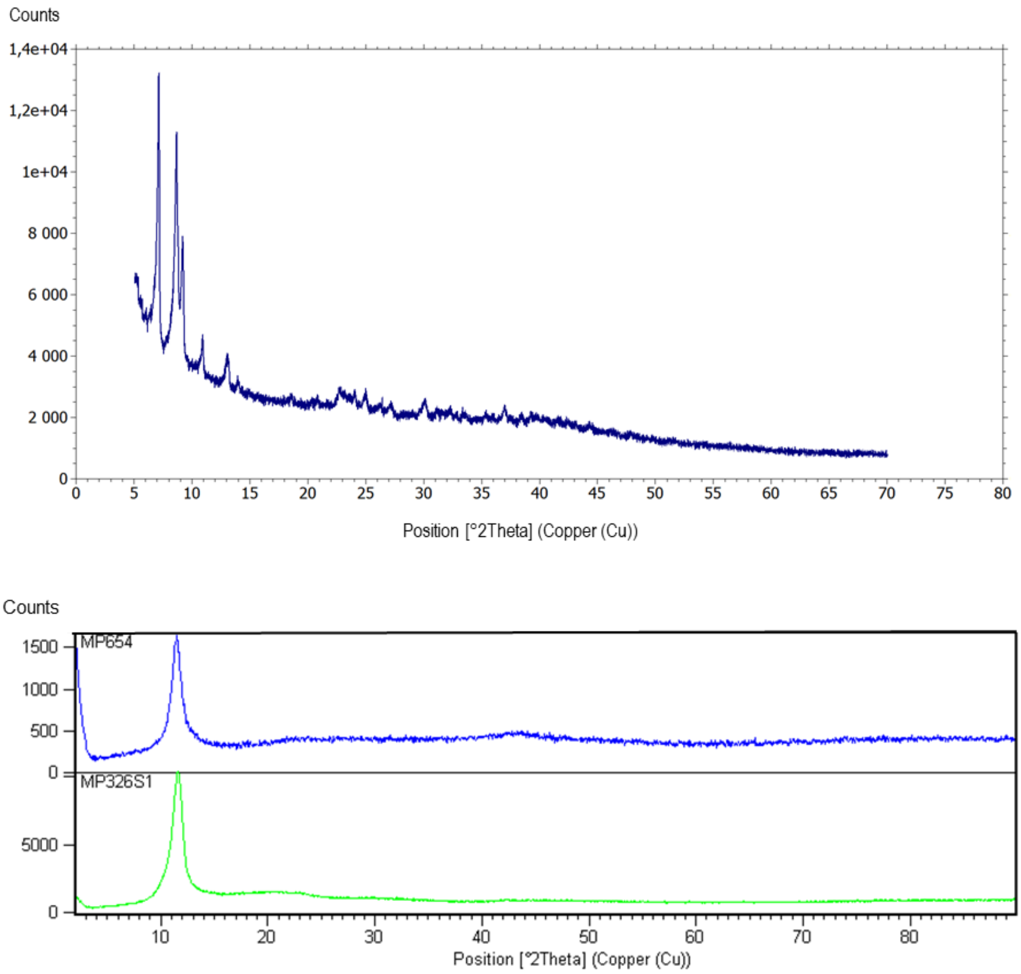

Figure S9. XRD patterns (from top to bottom) of $(TBA)_2[Mo_6I^i_8(O_2CCH_3)^a_6]$, $(TBA)_2Mo_6I^i_8$@GO and GO.

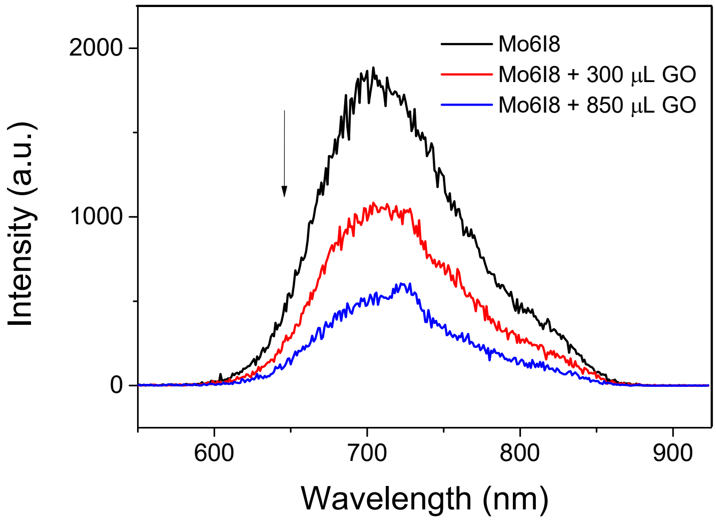

Figure S10. Steady state photoluminescence spectra (λ_{exc} = 345 nm) which depict the quenching of the $(TBA)_2[Mo_6I^i_8(O_2CCH_3)^a_6]$ luminescence (initial concentration 10^{-5} M in DMF) upon addition of increasing volumes of a stock dispersion of GO (2.5 mg/L) in DMF.

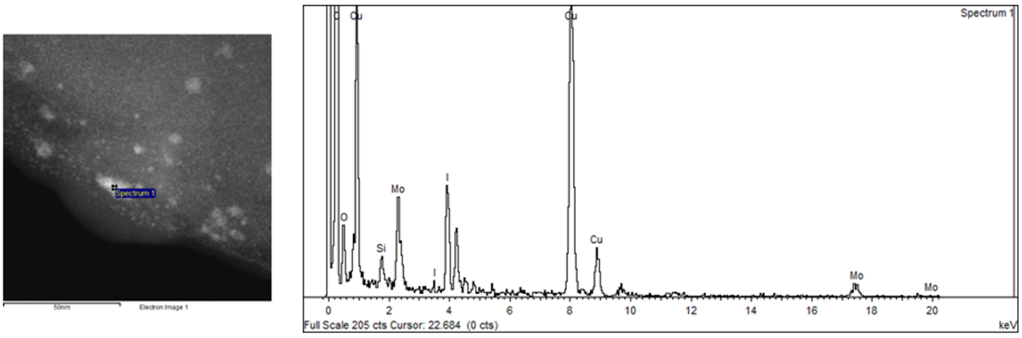

(**a**) (**b**)

Figure S11. Scanning transmission electron microscopy (STEM) image (a) and EDXA (b) of $(TBA)_2Mo_6I^i_8$@GO.

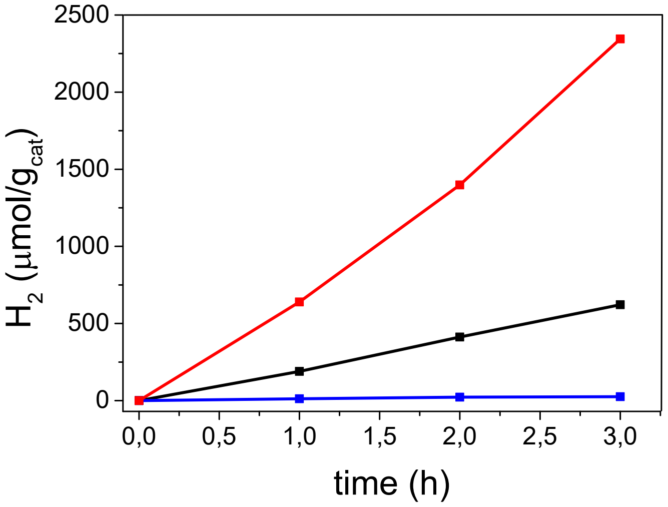

Figure S12. HER (µmol of H_2/g_{cat}) *vs* time plot by using the $(TBA)_2Mo_6I^i_8$@GO (11 mg, black line), GO (11 mg, blue line) and $(TBA)_2[Mo_6I^i_8(O_2CCH_3)^a_6]$ (0.54 mg, red line) catalysts in aqueous solution containing water/acetone/TEA mixture (50/45/5% v/v).

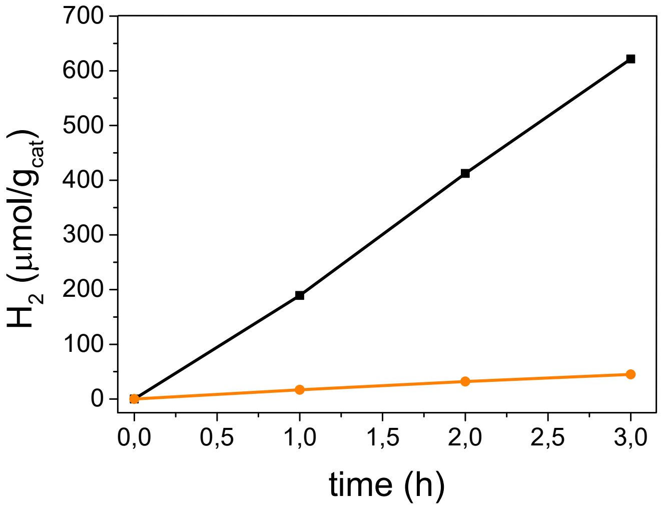

Figure S13. HER (µmol of H_2/g_{cat}) *vs* time plot by using the $(TBA)_2Mo_6I^i_8$@GO (11 mg, black line) and the recycled solid (11 mg, orange line) in aqueous solution containing water/acetone/TEA mixture (50/45/5% v/v).

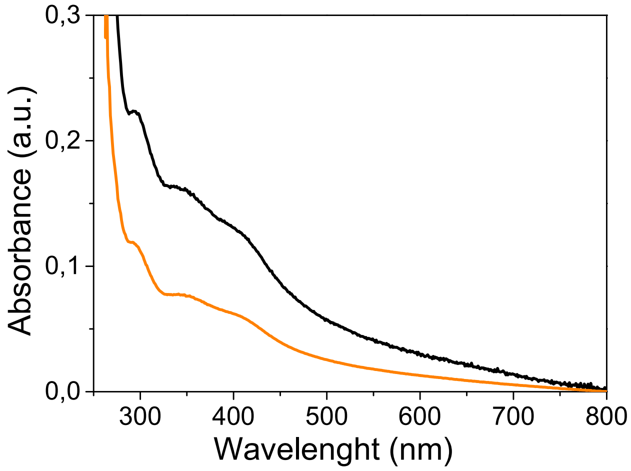

Figure S14. UV-vis spectra of the most characteristic bands of $(TBA)_2[Mo_6I^i_8(O_2CCH_3)^a_6]$ before (black line) and after (orange line) catalytic reaction under vapor phase conditions.

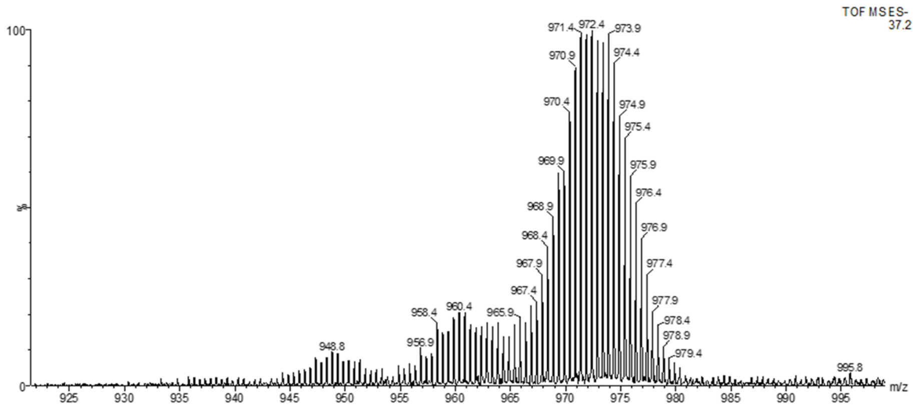

Figure S15. ESI mass generated molecular peaks (from right to left): $[Mo_6I^i_8(O_2CH_3)^a_6]^{2-}$, $[Mo_6I^i_7(O_2CH_3)^a_5Cl]^{2-}$ and $[Mo_6I^i_8(O_2CH_3)^a_5Br]^{2-}$ detected of a reaction sample after 24 h reaction time of the catalytic reaction in gas phase. The detection of two less intense peaks is ascribed to the presence of interferences in the electrospray source.

Calculation of the singlet and triplet excited states of the $[Mo_6I^i_8(O_2CCH_3)^a_6]^{2-}$ complex.

The energy of the $[Mo_6I^i_8(O_2CCH_3)^a_6]^{2-}$ complex singlet excited state ($E(S_1)$) was calculated from the energy level of the singlet ground state ($E(S_0)$) of the cluster, and adding to it the energy of the absorbed light, according to the equation (1). We have calculated the optical band onset (noted as E_g) and the onset potential of the first oxidation process (noted as E_{ox} in the absolute scale) of the $(TBA)_2[Mo_6I^i_8(O_2CCH_3)^a_6]$ compound.

$$E(S_1) = E(S_0) + E_g \quad (1)$$

E_g has been determined from the Tauc plot, which represents the square root of the absorption coefficient multiplied by photon energy, $(\alpha h\nu)^{1/2}$, as a function of photon energy $h\nu$. Figure S16 represents the absorption spectrum of $(TBA)_2[Mo_6I^i_8(O_2CCH_3)^a_6]$ and the linear extrapolation of the Tauc plot to the baseline to yield the onset of the optical absorption, E_g (3.15 eV).

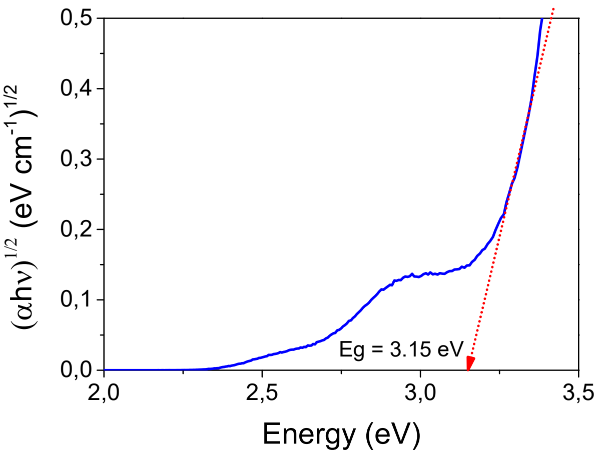

Figure S16. Tauc plot of $(TBA)_2[Mo_6I^i_8(O_2CCH_3)^a_6]$ from UV-vis spectrum registered in acetonitrile.

The onset potential is determined from the cyclic voltammogram (E_{onset} = 0.40 V *vs* Ag/Ag$^+$), which has been referenced to the SHE electrode (E_{onset}(SHE) = 0.599 V) [12,13]. The absolute onset potential (E_{ox}) has been calculated by applying the equation (2) to give a E_{ox} value of 5.039 V. The energy of S_0 is calculated from to $e \cdot E_{ox}$, being e the electron charge. If the unit of charge is -1, the $E(S_0)$ is obtained in electron volts (eV) which corresponds to -5.039 eV for $[Mo_6I^i_8(O_2CCH_3)^a_6]^{2-}$. The application of eq.1 determines the value of $E(S_1)$ as -1.889 eV.

$$E_{ox} = E_{onset}(SHE) + 4.44\ V \quad (2)$$

The energy of the $[Mo_6I^i_8(O_2CCH_3)^a_6]^{2-}$ complex triplet excited state ($E(T_1)$) is determined according to equation (3), from the energy level of the singlet ground state (S_0) of the cluster, and the optical band onset of the emission spectra (E'_g = 1.99 eV (in acetonitrile), see Figure S17), to give a $E(T_1)$ value of -3.049 eV. The emission maxima registered in solid state (699 nm) shows a slight hypsochromic shift with respect to the maxima in solution (705 nm), but the optical band onset is constant.

$$E(T_1) = E(S_0) + E'_g \quad (3)$$

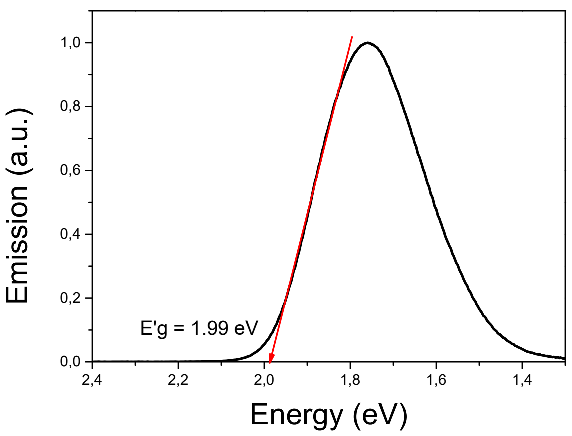

Figure S17. Emission spectrum of $(TBA)_2[Mo_6I^i_8(O_2CCH_3)^a_6]$ acquired in acetonitrile.

References

1. Recatalá, D.; Llusar, R.; Gushchin, A.L.; Kozlova, E.A.; Laricheva, Y.A.; Abramov, P.A.; Sokolov, M.N.; Gómez, R.; Lana-Villarreal, T. Photogeneration of Hydrogen from Water by Hybrid Molybdenum Sulfide Clusters Immobilized on Titania. *ChemSusChem* **2015**, *8*, 148–157, doi:10.1002/cssc.201402773.
2. Zhu, B.; Lin, B.; Zhou, Y.; Sun, P.; Yao, Q.; Chen, Y.; Gao, B. Enhanced photocatalytic H2 evolution on ZnS loaded with graphene and MoS2 nanosheets as cocatalysts. *J. Mater. Chem. A* **2014**, *2*, 3819–3827, doi:10.1039/C3TA14819J.
3. Zong, X.; Na, Y.; Wen, F.; Ma, G.; Yang, J.; Wang, D.; Ma, Y.; Wang, M.; Sun, L.; Li, C. Visible light driven H2 production in molecular systems employing colloidal MoS2nanoparticles as catalyst. *Chem. Commun.* **2009**, 4536–4538, doi:10.1039/B907307H.
4. Feliz, M.; Puche, M.; Atienzar, P.; Concepción, P.; Cordier, S.; Molard, Y. In Situ Generation of Active Molybdenum Octahedral Clusters for Photocatalytic Hydrogen Production from Water. *ChemSusChem* **2016**, *9*, 1963–1971, doi:10.1002/cssc.201600381.
5. Wang, M.; Han, X.; Zhao, Y.; Li, J.; Ju, P.; Hao, Z. Tuning size of MoS2 in MoS2/graphene oxide heterostructures for enhanced photocatalytic hydrogen evolution. *J. Mater. Sci.* **2018**, *53*, 3603–3612, doi:10.1007/s10853-017-1745-7.
6. Zhang, Y.; Zhang, Y.; Li, X.; Zhao, X.; Anning, C.; Crittenden, J.; Lyu, X. Photocatalytic water splitting of ternary graphene-like photocatalyst for the photocatalytic hydrogen production. *Front. Environ. Sci. Eng.* **2020**, *14*, 69, doi:10.1007/s11783-020-1248-7.
7. Zhang, Y.; Zhang, Y.; Li, X.; Dai, J.; Song, F.; Cao, X.; Lyu, X.; Crittenden, J.C. Enhanced Photocatalytic Activity of SiC-Based Ternary Graphene Materials: A DFT Study and the Photocatalytic Mechanism. *ACS Omega* **2019**, *4*, 20142–20151, doi:10.1021/acsomega.9b01832.
8. Sheldrick, G. A short history of SHELX. *Acta Crystallogr. Sect. A* **2008**, *64*, 112–122, doi:10.1107/S0108767307043930.
9. Sheldrick, G. Crystal structure refinement with SHELXL. *Acta Crystallogr. Sect. C* **2015**, *71*, 3–8, doi:10.1107/S2053229614024218.
10. Dolomanov, O. V; Bourhis, L.J.; Gildea, R.J.; Howard, J.A.K.; Puschmann, H. OLEX2: a complete structure solution, refinement and analysis program. *J. Appl. Crystallogr.* **2009**, *42*, 339–341, doi:10.1107/S0021889808042726.
11. Mikhaylov, M.A.; Abramov, P.A.; Komarov, V.Y.; Sokolov, M.N. Cluster aqua/hydroxocomplexes supporting extended hydrogen bonding networks. Preparation and structure of a unique series of cluster hydrates [Mo6I8(OH)4(H2O)2]·nH2O (n=2, 12, 14). *Polyhedron* **2017**, *122*, 241–246, doi:10.1016/j.poly.2016.11.011.
12. Mikhailov, M.A.; Brylev, K.A.; Abramov, P.A.; Sakuda, E.; Akagi, S.; Ito, A.; Kitamura, N.; Sokolov, M.N. Synthetic Tuning of Redox, Spectroscopic, and Photophysical Properties of {Mo6I8}4+ Core Cluster Complexes by Terminal Carboxylate Ligands. *Inorg. Chem.* **2016**, *55*, 8437–8445, doi:10.1021/acs.inorgchem.6b01042.
13. Trasatti, S. The absolute electrode potential: an explanatory note (Recommendations 1986). *Pure Appl. Chem.* **1986**, *58*, 955–966, doi:10.1351/pac198658070955.
